# Multi-platform profiling reveals host- and cell -type-specific pseudorabies virus gene expression

**DOI:** 10.1038/s41598-026-45990-4

**Published:** 2026-04-01

**Authors:** Balázs Kakuk, Zsolt Csabai, Zoltán Deim, Gábor Torma, Ádám Fülöp, Gergely Ármin Nagy, Virág Éva Dani, Dóra Tombácz, Zsolt Boldogkői

**Affiliations:** 1 Department of Medical Biology, Albert Szent-Györgyi Medical School, University of Szeged, Szeged, Hungary; 2 Department of Physiology, Anatomy and Neuroscience, Faculty of Science and Informatics, University of Szeged, 6726 Szeged, Hungary

**Keywords:** Computational biology and bioinformatics, Genetics, Molecular biology

## Abstract

Pseudorabies virus (PRV) is an alphaherpesvirus that follows a conserved immediate-early → early → late transcriptional cascade, yet how this program adapts to diverse cell types is unclear. We profiled PRV transcription in four permissive cell lines—two epithelial (porcine kidney PK-15 and rat kidney NRK), one glial (rat glioma C6), and one neuron-like (rat PC-12)—at six time points (1–12 h post-infection). Host-dependent differences peaked early in infection, particularly for the regulators *ie180*, *ep0*, and *us1*. *ie180* showed strong species bias, with high expression in PK-15 but minimal in rodent lines, whereas *ep0* and *us1* varied quantitatively by cell type, with C6 and PC-12 showing marked early *us1* activation. Combining long-read direct cDNA and direct RNA sequencing with 5′-capped CAGE-seq resolved viral transcription boundaries and identified 94 previously unannotated transcripts, including 5′ UTR isoforms, polygenic RNAs, and noncoding transcripts. Differential transcript usage analysis revealed extensive isoform remodeling across conditions, with late infection showing shifts from long or polygenic isoforms toward shorter forms. Together, these results provide the first multi-host, isoform-resolved temporal atlas of PRV transcription and show that the canonical cascade is conserved yet quantitatively tuned by host species and cell-type background.

**Supplementary Information:**

The online version contains supplementary material available at 10.1038/s41598-026-45990-4.

## Introduction

Pseudorabies virus (PRV; *Suid herpesvirus 1*) is an alphaherpesvirus of swine and the causative agent of Aujeszky’s disease, widely used as a model for herpesvirus molecular pathogenesis and as a trans-neuronal tracer for mapping multisynaptic neural circuits^1–6^. PRV is closely related to varicella-zoster virus (VZV), equid alphaherpesvirus 1 (EHV-1) and bovine alphaherpesvirus 1 (BoHV-1)^7–9^, and carries a ~ 143 kb double-stranded DNA genome encoding ~ 150 proteins and several non-coding RNAs (ncRNAs)^4,5^, including the CTO-S^10^ and NOIR1^11^.

The transcriptional cascade of alphaherpesviruses unfolds in three kinetic waves—immediate-early (IE), early (E), and late (L)—driven by host RNA polymerase II^12^. Across alphaherpesviruses, a conserved regulatory module composed of the *icp4*/*ie180*, *icp0*/*ep0*, and *icp22*/*us1* homologs coordinates the viral transcriptional cascade, integrating DNA binding, chromatin modulation, and RNA polymerase II regulation^13,15^. In PRV, this module is deployed with distinct kinetics: *ie180* functions as a potent DNA-binding transactivator, whereas *ep0* and *us1*—classified as IE in HSV-1—are expressed with E kinetics^13,14^. Recent nascent-transcript profiling in HSV-1 has further shown that these IE regulators initially impose a transient, genome-wide repression of RNA polymerase II activity before subsequent gene-class–specific activation, reframing the cascade as a process of controlled derepression rather than simple activation^16^. E genes are required for DNA replication, whereas L genes mainly encode structural components needed for virion assembly, entry, and egress^12,15^. Although a coarse IE → E → L order is established, the molecular triggers for the E-to-L transition—and how they vary across cell types and hosts—remain incompletely defined. PRV can also persist latently in sensory neurons of the trigeminal ganglion and periodically reactivate^4,5^.

Early studies on HSV-1 showed that IE gene expression can differ substantially between cell types: the transcriptional activator ICP0, a homolog of PRV EP0, accumulates rapidly in permissive epithelial cells but only weakly in neuronal or quiescent models^17,18^. Similar regulatory divergence occurs in PRV: glucocorticoid signaling enhances *ie180* transcription in neuronal-like Neuro-2A cells but suppresses it in epithelial PK-15 cells through opposing actions of the glucocorticoid receptor and Oct-1^19^.

Cell type influences viral transcriptome kinetics and composition. In HSV-1 infection, PC-12 neuron-like cells show altered IE regulation (ICP0, ICP4) and LAT accumulation^18,20^, while C6 glioma cells require interferon pathway suppression for efficient replication^21^. In PRV-infected PK-15 cells, viral transcription is dynamically coordinated with host chromatin via RUNX1-mediated promoter interactions^22^. PRV replicates efficiently in porcine epithelial cell culture systems (including PK-15), whereas infection outcomes in non-natural host cells can be restricted in a host- and cell-type–dependent manner, including through innate interferon-mediated restriction^23,24^.

Consistent with this, Yin and colleagues demonstrated that distinct rat epithelial cell types mount differential type I and type III interferon responses to PRV infection, resulting in cell-type–specific antiviral activity and viral replication dynamics^25^. In swine, PRV typically causes mild respiratory disease followed by neuronal latency, whereas rodent infections are generally fatal^26^. These cell-type– and species-specific outcomes are consistent with differential innate immune restriction and neuronal permissiveness^23–26^.

To resolve the molecular basis of these host-dependent differences, high-resolution transcriptomic approaches are required. In HSV-1, time-resolved RNA sequencing revealed sequential activation of IE, E, and L gene classes, with transcript levels fluctuating dynamically over time even within the same kinetic class^27,28^. Similar approaches in human cytomegalovirus (HCMV) identified a more complex, multi-phase program extending beyond the classical IE/E/L pattern^29^. However, these studies lacked resolution to distinguish overlapping transcription units or closely related RNA isoforms. Recent long-read RNA sequencing (lrRNA-seq) technologies, such as Pacific Biosciences SMRT and Oxford Nanopore Technologies (ONT) cDNA and direct RNA sequencing (dRNA-seq), overcome the limitations of short-read approaches by capturing complete viral transcripts and accurately mapping transcription-start sites (TSSs) and transcription end sites (TESs)^30,31^. Although native RNA sequencing underrepresents true 5′ ends due to technical bias^32^, integrating Cap Analysis of Gene Expression sequencing (CAGE-seq) with direct cDNA sequencing addresses this by mapping capped 5′ ends at base-pair resolution and refining promoter annotations^33^.

When applied to alphaherpesviruses, these approaches have revealed the multilayered architecture and dynamics of alphaherpesvirus gene expression^9,33,34^. In BoHV-1, time-course Nanopore sequencing showed that individual promoters generate multiple TSS variants whose abundances shift during infection, along with overlapping and read-through isoforms that change over time^14^. In EHV-1, integrated CAGE-seq and direct cDNA sequencing revealed numerous co-expressed RNA isoforms with distinct start and end sites; long read-through transcripts are progressively replaced by shorter forms during infection^35^. These studies demonstrated widespread dynamic changes in promoter usage, TSS/TES variation, and isoform remodeling—features obscured in short-read datasets. Despite such progress, it remains unclear how much of PRV’s transcriptional flexibility is driven by host species versus cell type differences, and which regulatory features are truly conserved across distinct host and cell-type environments.

## Results

### Integrated sequencing reveals PRV transcript diversity and novel isoforms

In this study, we profiled PRV transcription in four permissive cell lines—PK-15 (porcine kidney epithelium), NRK (rat kidney epithelium), C6 (rat glioma), and PC-12 (rat neuron-like cells). We used three complementary sequencing approaches—including isoform-resolved direct cDNA sequencing (dcDNA-seq) and dRNA-seq on ONT MinION platform, and 5′-capped CAGE-seq on Illumina MiSeq platform—to compare PRV transcripts across host species and cell types and annotate novel viral RNAs.

Direct cDNA sequencing was performed at six infection time points (1, 2, 4, 6, 8, and 12 hpi), with three biological replicates per time point. Each cell line was represented by one pooled CAGE-seq library to capture capped 5′ ends. Direct RNA sequencing libraries were generated for PK-15, C6, and PC-12 cells to validate full-length transcripts and 3′ end coordinates across representative epithelial and neuronal/glial backgrounds. An NRK dRNA-seq library was not generated, as preliminary analyses indicated that splicing and TES usage patterns in NRK were highly concordant with those observed in C6, PC-12, and PK-15, with no cell-type–specific splicing differences detected. Accordingly, an additional rat epithelial dRNA-seq library was considered unlikely to yield further biological insight.

In total, the dcDNA-seq libraries yielded ~ 52 million reads, of which 4.1 million mapped unambiguously to the PRV genome. The CAGE-seq libraries contained ~ 9 million tags per cell line, and the dRNA-seq libraries ~ 1.6 million reads per line. Although smaller in scale, these datasets were used to validate transcript boundaries and intron junctions with high precision (Supplementary Table S1).

A unified PRV transcript reference was generated by integrating a previously published transcript collection^37,38^; transcripts not matching this reference were retained as novel only if their 5′ ends were supported by either dcDNA-seq replicates or pooled CAGE-seq libraries, and their 3′ ends were validated by dcDNA-seq or native RNA reads. The transcripts were then assigned to their parent genes, structurally classified, and quantified across infection stages and cell lines (Supplementary Tables S2–S3). Each transcript had to be present in at least three different samples with a total minimum count of five to be designated as detected. This dual requirement excludes sporadic reads from template switching, incomplete reverse transcription, or stochastic sampling while remaining sensitive to genuine low-abundance transcripts. Although no universal standard exists for long-read RNA-seq, our threshold is comparable to or more stringent than thresholds applied in similar viral transcriptome studies.

Because 5′-truncated transcripts may arise from incomplete reverse transcription, internal priming during cDNA synthesis, RNA degradation, or template switching artifacts inherent to long-read sequencing (LRS) technologies, these categories were subjected to further stringent filtering. Transcripts were retained only if both termini were supported by dRNA-seq reads (TES and TSS) and their 5′ end was independently confirmed by CAGE-seq. In addition, they had to contribute at least 25% of the total expression of their parent gene in at least one sample. Following prior usage, we refer to 5′-truncated, in-frame isoforms that encode N-terminally shortened proteins and are coterminal with their canonical coding sequence as putative embedded gene transcripts^30,32,33^. Although they technically encode independent ORFs, we annotated them under their parent genes for consistency; therefore, throughout this study, the term “isoforms” also encompasses these putative transcripts. This additional stringent filtering (and the minimum prevalence and absolute count filtering) was applied to the reference set as well, and as a result, a substantial number of transcripts remained in the final atlas. Of these, 214 originated from the earlier reference set, and 94 represented newly detected isoforms supported by our multi-platform data.

### LRS reveals TSS diversity and widespread polygenic transcription

Comprehensive long-read profiling uncovered diverse previously unannotated transcripts and regulatory features, including alternative TSSs and terminators, polygenic RNAs, spliced and ncRNA molecules, as well as complex transcripts (RNA molecules containing at least one ORF in an antiparallel orientation). Among the newly detected RNAs, 12 were long 5′ UTR isoforms and two were 3′-short UTR variants, including one each from the *us1* and *ul3* genes. We also identified a novel but rare splice site in *ul42* and two spliced transcripts carrying this intron that differed only in their 5′ ends. To minimize artifactual misannotation, we applied additional stringent filtering to 3′- and 5′-short UTR isoforms, ncRNA transcripts arising from 3′ truncation, and putative 5′-truncated mRNAs. After filtering, ncRNA transcripts and 5′-short UTR isoforms that passed quality control accounted for five and three transcripts, respectively. In addition, 25 putative embedded gene transcripts were annotated, these harbored only the truncated version of their parent ORF, and 16 additional putative polygenic transcripts contained a complete downstream ORF in addition to the truncated upstream ORF.

**Fig. 1:**
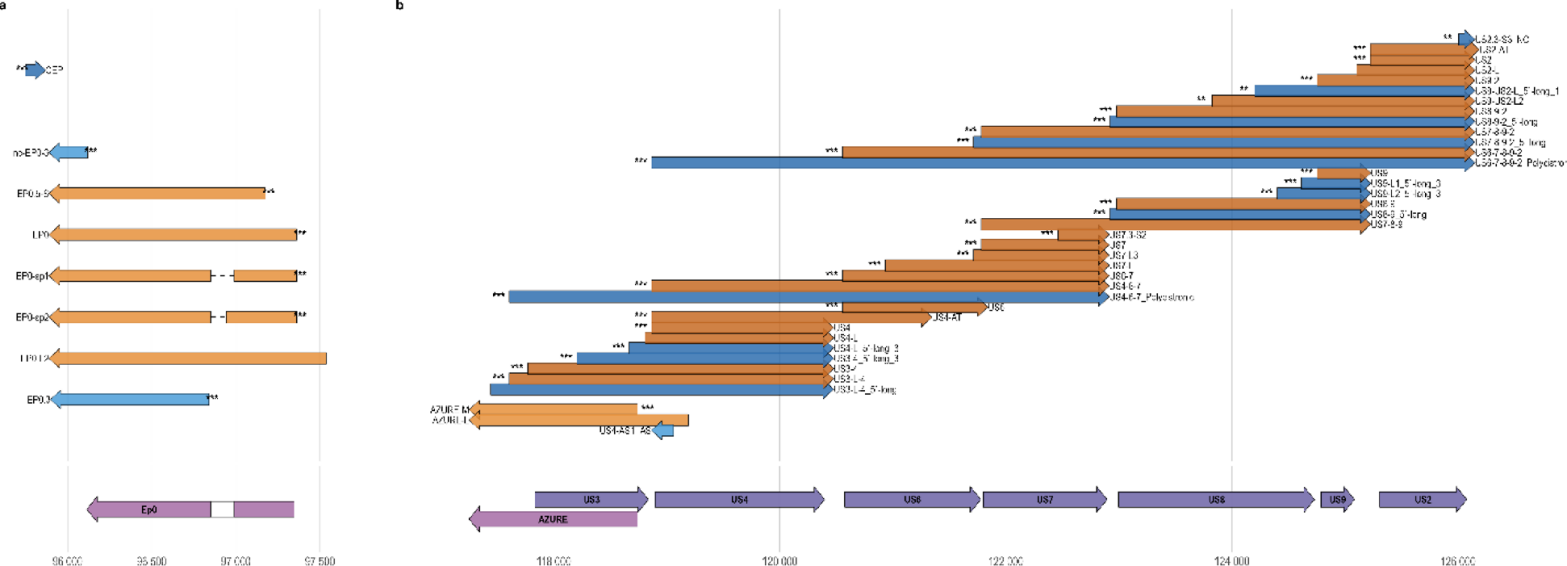
Transcript architecture of key regulatory regions and *us1* isoform diversity. (**a**–**b**) Transcript architecture of the PRV *ie180* (**a**) and US (**b**) regions. Genome coordinates are shown on the horizontal axis, with annotated coding sequences (CDSs) marked in red, the non-coding gene AZURE in green, and OriS-I in blue. Transcript models (bottom) depict annotated and putative transcripts, colored according to whether they were newly annotated in this work (blue) or previously described (orange).

We identified CEP, a low-abundance transcript convergent to *ep0* (Fig. 1a), which appears to be a 3′-truncated version of the long latency transcript. Novel polygenic transcripts (8), 5′-long (6) and 5′-short (1) UTR variants of previously described polygenic RNAs, and three complex transcripts (polygenic transcripts carrying complete ORFs in the antisense orientation) indicate extensive transcriptional read-through between neighboring and distal genes. Several polygenic transcripts spanned multiple genes, including the longest transcript yet observed (7288 bp), linking UL16–UL11 in a single RNA, resulting from novel read-through between *ul16* and *ul14*. Additionally, the US region harbored unusually long polygenic RNAs, including US3–US4–US6–US7 and US6–US9, the latter spanning nearly the entire US cluster and representing one of the longest PRV transcripts overall (Fig. 1b). We detected five antisense transcripts as well, including a short antisense transcript in the *us4* region (Fig. 1b).

Longer transcripts are more likely to reflect bona fide RNA molecules, as their extended length makes it unlikely that they originate from artefactual truncation. Accordingly, these RNAs are considered genuine transcriptional products, although the exact positions of their 5′ or 3′ ends may not always be fully resolved. In contrast, transcripts truncated at either the 5′ or 3′ end cannot be unambiguously distinguished from products of RNA processing or technical artefacts. Such truncations may arise biologically through intracellular RNA cleavage and, in some cases, subsequent recapping, or may be introduced during library preparation and reverse transcription, with 5′ truncation being particularly common. For this reason, non-truncated transcripts are reported in Supplementary Table S3A, whereas transcripts exhibiting 5′- or 3′-end truncation were classified as putative and are reported separately (Supplementary Table S3B). Having defined the full transcriptome structure, we next examined how transcriptional activity evolved across infection and differed among hosts.

### Transcript abundance varies across host species and cell types

**Fig. 2:**
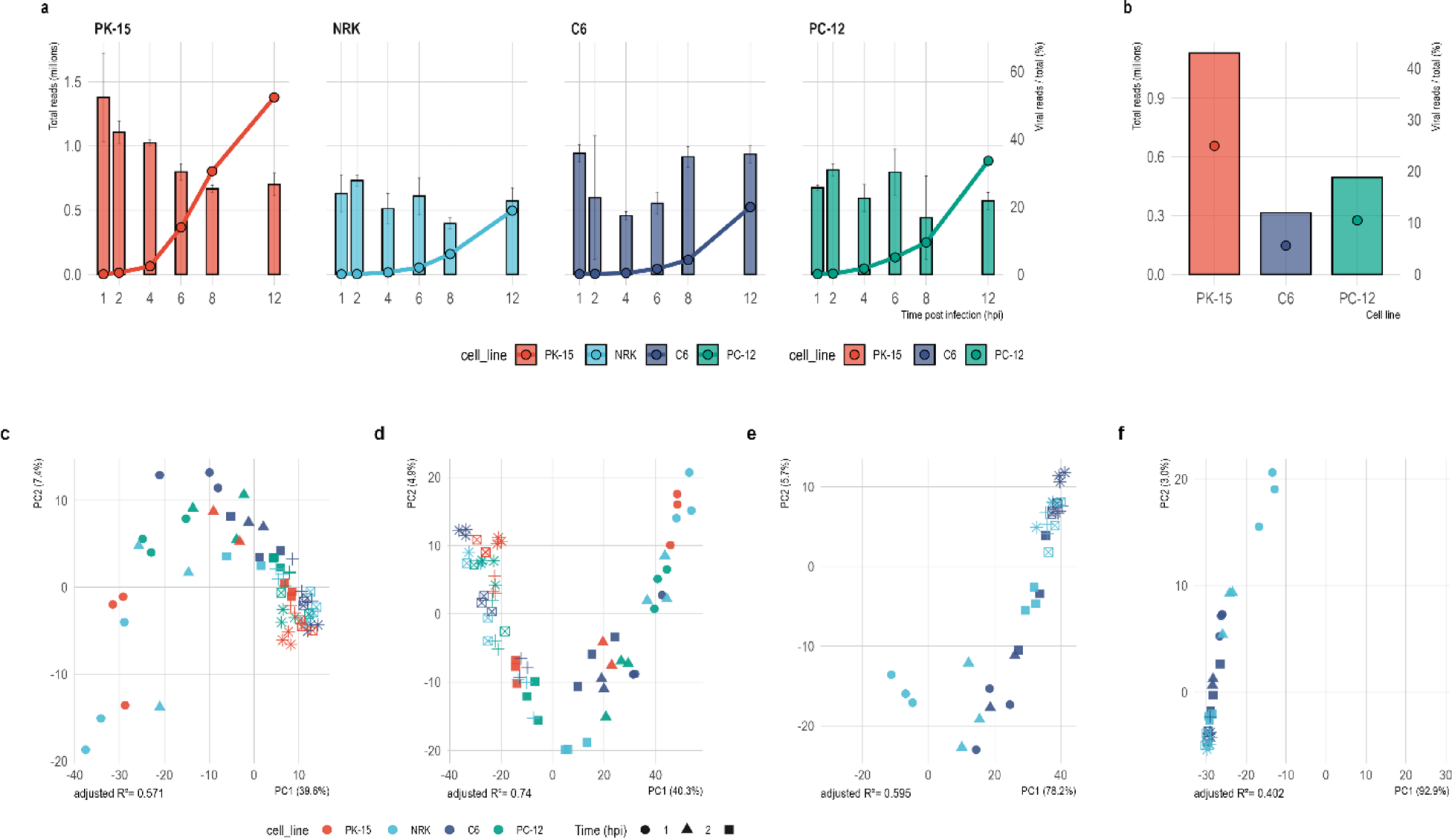
Sequencing depth and viral read ratios across cell lines and library types. (**a**) Kinetics of PRV transcripts detected by dcDNA-seq. Bars show total sequencing reads (millions) per time point (mean ± SD across replicates). The overlaid line and points indicate the proportion of viral reads relative to all reads (secondary y-axis). (**b**) Direct RNA sequencing data from mixed-time-point libraries. Bars show total read output per cell line, while points denote the viral/total read ratio on the secondary axis. (**c**–**f**) Principal component analysis (PCA) performed on (**c**) gene-level counts, (**d**) transcript-level abundances, (**e**) TES-level and (**f**) TSS-level distributions. Colors denote cell lines and shapes denote infection time points.

Both long-read sequencing approaches (dcDNA-seq and dRNA-seq) revealed pronounced host-dependent differences in viral transcript abundance. PK-15 cells consistently produced the highest viral read fractions, whereas rodent-derived cell lines showed lower overall levels. In dcDNA-seq (Fig. 2a), viral RNA increased sharply from 6 hpi onward across all cell lines, albeit with distinct magnitudes. By 12 hpi, PK-15 cells converted more than half of total reads to viral RNA (~ 52%), while PC-12 reached ~ 34%, and NRK and C6 plateaued at ~ 19–20%. Thus, PC-12 exhibited a delayed yet substantial late-stage accumulation of viral transcripts, in contrast to the more modest late-phase levels observed in NRK and C6. Complementary dRNA-seq libraries, generated from pooled mixed time points (Fig. 2b), recapitulated the same rank order of viral output, with PK-15 showing the highest mean viral fraction (~ 25%), followed by PC-12 (~ 10%) and C6 (~ 6%). Although absolute values were lower due to pooling across early and late stages, the concordant host-specific trends between dcDNA- and dRNA-seq indicate that these differences reflect genuine biological variation rather than platform-specific bias.

To determine whether these host-associated differences reflected qualitative changes in transcript structure or primarily quantitative shifts, we compared isoform presence across species, cell lines, and sequencing platforms. Of 308 detected PRV transcript isoforms, only one was exclusive to the porcine PK-15 line and five were restricted to rat-derived lines, while cell-type–restricted isoforms were similarly rare (four epithelial-only and three neural/glial-only transcripts). Regarding platform specificity, no isoform was uniquely supported by dRNA-seq, while 71 were detected only in dcDNA libraries. Thus, the PRV transcriptome architecture was largely conserved across hosts and platforms, and host-dependent variation predominantly reflected differences in transcript abundance rather than the emergence of lineage-specific isoforms. Supplementary Figure S1 visualizes these overlaps using an UpSet plot, demonstrating that the vast majority of detected PRV isoforms are shared across host backgrounds and sequencing platforms, with only a small subset restricted to individual cell lines or library types.

**Fig. 3:**
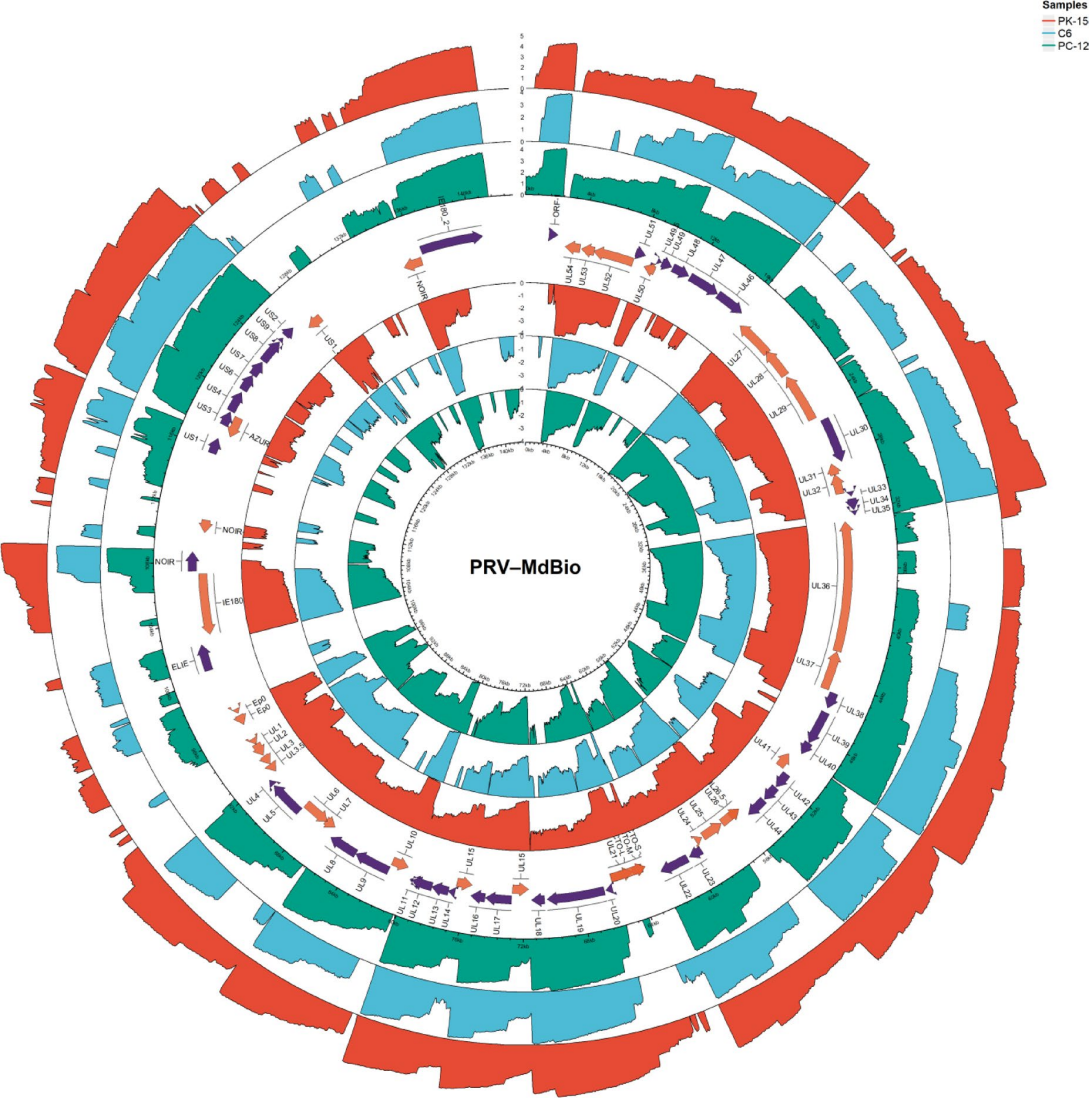
PRV direct RNA coverage across cell lines in mixed-time-point libraries. Concentric rings show genome-wide, strand-specific PRV coverage for PK-15, PC-12, and C6 cells, with reads from all sampled time points pooled per cell line. Coverage is plotted as log₁₀(reads + 1) and displayed using a common y-axis scale across rings within the figure to enable comparison of relative genomic coverage patterns. Tracks on the outside correspond to the plus strand, and tracks on the inside correspond to the minus strand. Ring order and colors follow the legend. Because direct RNA libraries were generated from pooled samples normalized by total polyadenylated RNA input rather than viral RNA abundance, early infection stages with low viral read ratios contribute proportionally less to overall coverage. Consequently, the profiles predominantly reflect late and early–late transcriptional output.

Beyond isoform-level comparisons, genome-wide coverage profiles further illustrated the host-dependent magnitude of viral transcription. Genome-wide coverage from mixed–time-point dRNA-seq libraries was dominated by late and early–late transcription across all examined cell lines (Fig. 3). In PK-15, PC-12, and C6 cells, the highest mean contributions originated from large late-gene clusters, including *ul33–34–35, ul24–25–26–26.5, ul48–47–46, ul14–13–12–11, us3–4,* and *cto*, each accounting for substantial fractions of total viral reads. This pattern was consistent across hosts despite quantitative differences in overall coverage magnitude, with PK-15 exhibiting the strongest cumulative signal, followed by PC-12 and C6.

In contrast, IE and E regulatory genes contributed only a minor proportion of total dRNA signal in all cell lines. The *ie180*, *ep0*, and *us1* showed uniformly low mean abundances (typically < 1% of viral reads per gene), despite their essential regulatory roles. This reflects the mixed–time-point pooling strategy used for direct RNA sequencing: early infection samples, in which viral transcripts represent only a small fraction of total cellular RNA (Fig. 2b), contribute proportionally less to the pooled libraries. As a result, transcripts expressed primarily at 1–2 hpi are diluted relative to late and early–late genes, which dominate viral RNA output at later stages. Thus, the circos coverage profiles primarily capture the spatial organization and host-dependent magnitude of late-phase PRV transcription rather than early regulatory kinetics.

### Kinetic modeling reveals conserved timing but host-dependent amplitudes

For kinetic characterization, viral transcription was quantified at TSS and TES levels in each dcDNA-seq library across all six time points and three biological replicates per cell line; all values were normalized to total viral read counts. Differential gene expression at these boundaries was assessed using summed read counts from transcripts overlapping each TSS or TES and carrying the appropriate 5′ or 3′ adapter. Temporal trends were modeled with moanin spline interactions after TMM normalization, and additional models captured host- and cell-type-dependent effects (see Methods). In parallel, cumulative transcriptional output at each TSS and terminator was quantified using the area under the expression–time curve (AUC), and isoform-level differences were tested with DRIMSeq + stageR to assess time-, host-, and cell-type-dependent effects on transcript usage.

**Fig. 4:**
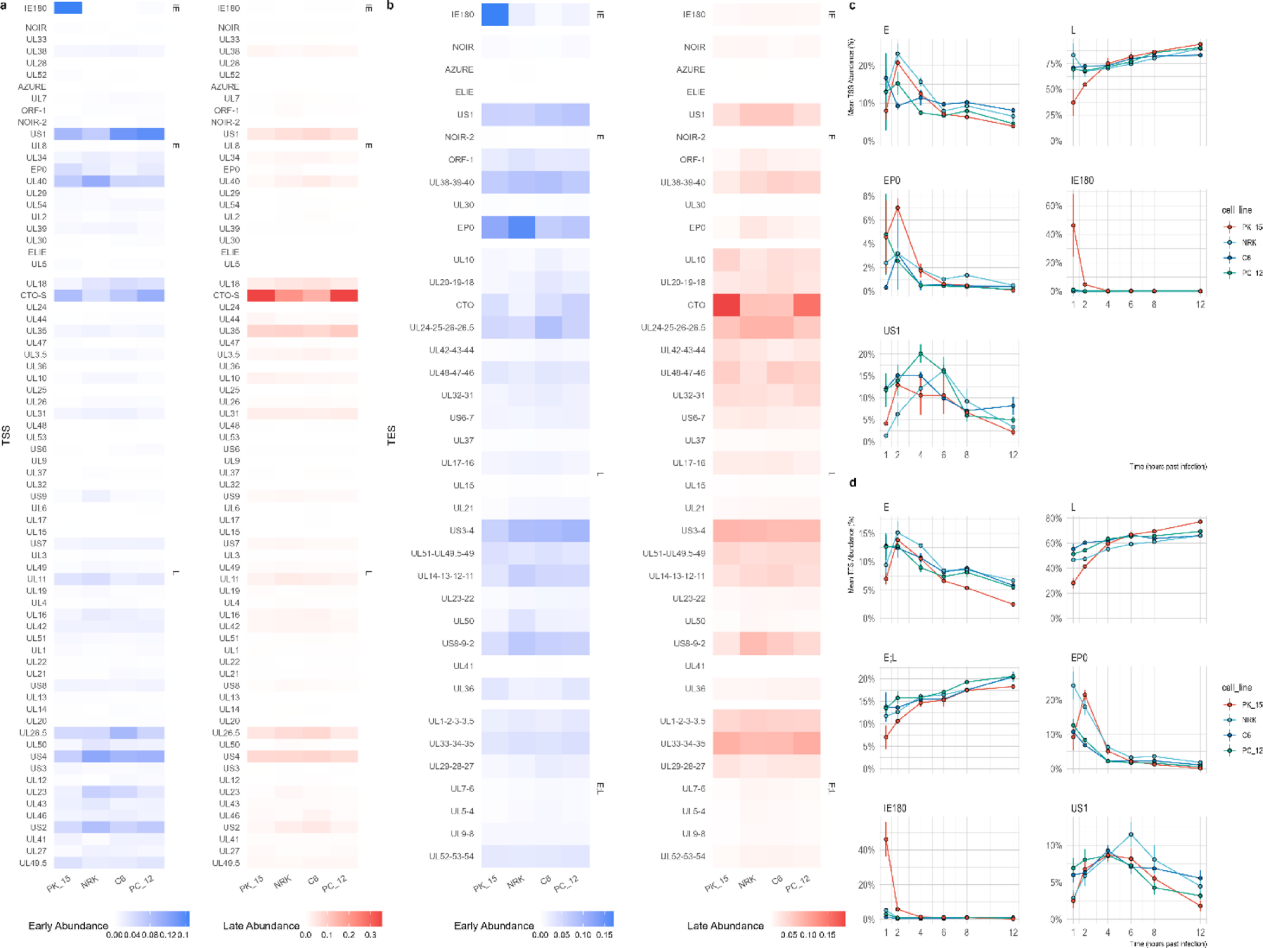
Early- and late-stage viral gene activity across cell lines based on TSS and TES usage. (**a**, **b**) Heatmaps of mean normalized viral gene abundance during early (1–4 hpi) and late (8–12 hpi) infection windows, based on transcription start site (TSS, **a**) and transcription end site (TES, **b**) quantifications. Each row represents a viral gene, ordered by its median peak time across all cell lines (early to late), and each column corresponds to one host background (PK-15, NRK, PC-12, C6). Blue and red indicate higher early and late abundances, respectively, while white denotes low or missing values. The TSS-based map captures promoter-driven initiation patterns, and the TES-based map reflects transcript completion and accumulation. (**c**) TSS kinetics of the regulatory triad (*ie180*, *ep0*, *us1*) alongside the summed abundances of early and late genes. Line plots show the proportion of viral reads initiating at each triad gene across PK-15, NRK, PC-12, and C6 cells. *ie180* dominates early in PK-15, *ep0* peaks at 1–2 hpi across hosts, and *us1* rises at mid-phase with strong expression in neuronal and epithelial lines. (**d**) TES kinetics of the regulatory triad together with the summed abundances of early and late genes. Line plots show TES usage as a share of viral reads. TES profiles mirror TSS dynamics, with early *ie180* dominance in PK-15, robust *ep0* peaks in both PK-15 and NRK, and mid-phase *us1* expression across all cell lines.

Quantitative comparison of total transcriptional output across kinetic classes at the TSS level (Fig. 4a and c) showed that IE and E TSSs reached their maximal activity between 1 and 4 hpi in all four cell lines. Although the temporal order was conserved, the amplitude differed markedly: PK-15 and NRK exhibited much stronger early activation than the neural/glial lines, which showed a weaker and slightly flatter early response. Late-start TSSs increased steadily from 4 hpi onward and became dominant by 12 hpi, particularly in the epithelial backgrounds.

TES-based profiles mirrored these kinetics (Fig. 4b and d). Early infection was characterized by strong termination at regulatory loci, followed by progressive accumulation of late structural coterminal units. Several of these—such as the *ul31*, *ul33*–*ul35*, and *ul51*–*ul49*.5–*ul49* regions—showed sustained increases up to 12 hpi, especially in PK-15 and, to a lesser extent, NRK. These data indicate that while the timing of TSS and terminator activation is broadly conserved, the amplitudes of the corresponding transcriptional programs diverge substantially across host backgrounds.

Complementing the kinetic modeling results, principal component analysis of viral gene-, transcript-, TES-, and TSS-level abundances revealed a conserved temporal trajectory across host cell lines (Fig. 2c–f). In all layers, PC1 primarily reflected infection time, while PC2 captured host-specific variation. The temporal signal was strongest at the transcript level (adjusted R2 ≈ 0.74 for PC1), followed by gene- and TSS-level profiles, whereas TES-level variation showed a comparatively weaker association with time (adjusted R2 ≈ 0.35–0.40). These results indicate that host-dependent modulation of viral transcription is evident across regulatory layers but is most clearly resolved at the transcript level.

### The ie180–ep0–us1 triad is conserved but host-tuned

Because genome-wide TSS analysis indicated early host-dependent divergence, we next assessed how the three principal regulators of the PRV transcriptional cascade—*ie180*, *ep0*, and *us1*—behaved across the four host backgrounds during the earliest stages of infection. TSS and TES kinetic summaries confirmed strong early activation at these loci at 1–2 hpi, whereas structural TSSs remained largely inactive at these time points, consistent with pre-replicative kinetics. Although the temporal sequence of activation was conserved across hosts, moanin time-course modeling detected significant host-associated quantitative differences for these regulators across organism and/or cell-line contrasts.

The* ie180* exhibited the strongest species-associated divergence. In PK-15 cells, a pronounced TSS burst occurred at 1 hpi, representing the highest single TSS amplitude observed at this stage. In contrast, all three rodent-derived lines showed markedly lower *ie180* initiation at 1 hpi. By 2 hpi, *ie180* output had already declined substantially in PK-15 and remained comparatively low in NRK, C6, and PC-12 during the early phase. TES-level abundances mirrored this pattern. Organism-level modeling confirmed a significant species effect across the time-course (q < 0.05).

To determine whether the weak *ie180* signal in rodent cells reflected delayed onset rather than reduced amplitude, we examined extended early TES measurements in PC-12 (0.5–2.5 hpi). *ie180* transcripts were detectable at 0.5 hpi but remained low throughout this window, without evidence of a shifted or delayed peak relative to PK-15. This supports a genuinely reduced and transient *ie180* response in the neural background rather than a temporal displacement.

The *ep0* was activated early across all hosts but exhibited a broader quantitative distribution. At 1 hpi, *ep0* TSS activity was lowest in C6, intermediate in NRK, and highest in PC-12 and PK-15, with PC-12 slightly exceeding PK-15 at this time point. By 2 hpi, PK-15 showed the strongest *ep0* TSS amplitude. TES abundances were generally higher than TSS values and displayed host-specific ordering: NRK exhibited the highest TES signal at 1 hpi, whereas PK-15 exceeded all other lines at 2 hpi. Organism-level modeling detected significant species-associated divergence across the time-course.

The *us1* activation occurred later than *ie180* and *ep0*, reaching peak abundance at mid-early time points (4–6 hpi), and displayed the opposite host preference. Neural and glial lines (C6 and PC-12) showed stronger early TSS activity than PK-15 and NRK, with detectable signal at 1 hpi and further increase by 2 hpi. PK-15 showed intermediate activation, and NRK remained comparatively lower during early infection. TES-level abundances followed the same relative ordering. In contrast to *ie180* and *ep0*, *us1* showed stronger cell-type–associated effects than species-level effects in time-course modeling.

Extended early PC-12 TES measurements demonstrated sustained *us1* presence throughout 0.5–2.5 hpi, consistent with early activation in neural backgrounds. Across all hosts, the temporal hierarchy of the regulatory cascade was conserved: *ie180* peaked earliest, followed by *ep0*, with *us1* rising slightly later. Host-dependent effects primarily altered the amplitude and relative balance of regulatory output rather than the order of activation. PK-15 displayed a dominant early *ie180* burst and strong *ep0* amplification, whereas rodent lines exhibited attenuated *ie180* output and relatively stronger *us1* activation. These findings indicate that the *ie180*–*ep0*–*us1* triad is kinetically conserved but quantitatively tuned by host species and tissue background. Supplementary Table S4 shows the mean abundances of the regulatory triad across the cell lines.

### TSS usage separates porcine from rodent hosts and epithelial from other cells

**Fig. 5:**
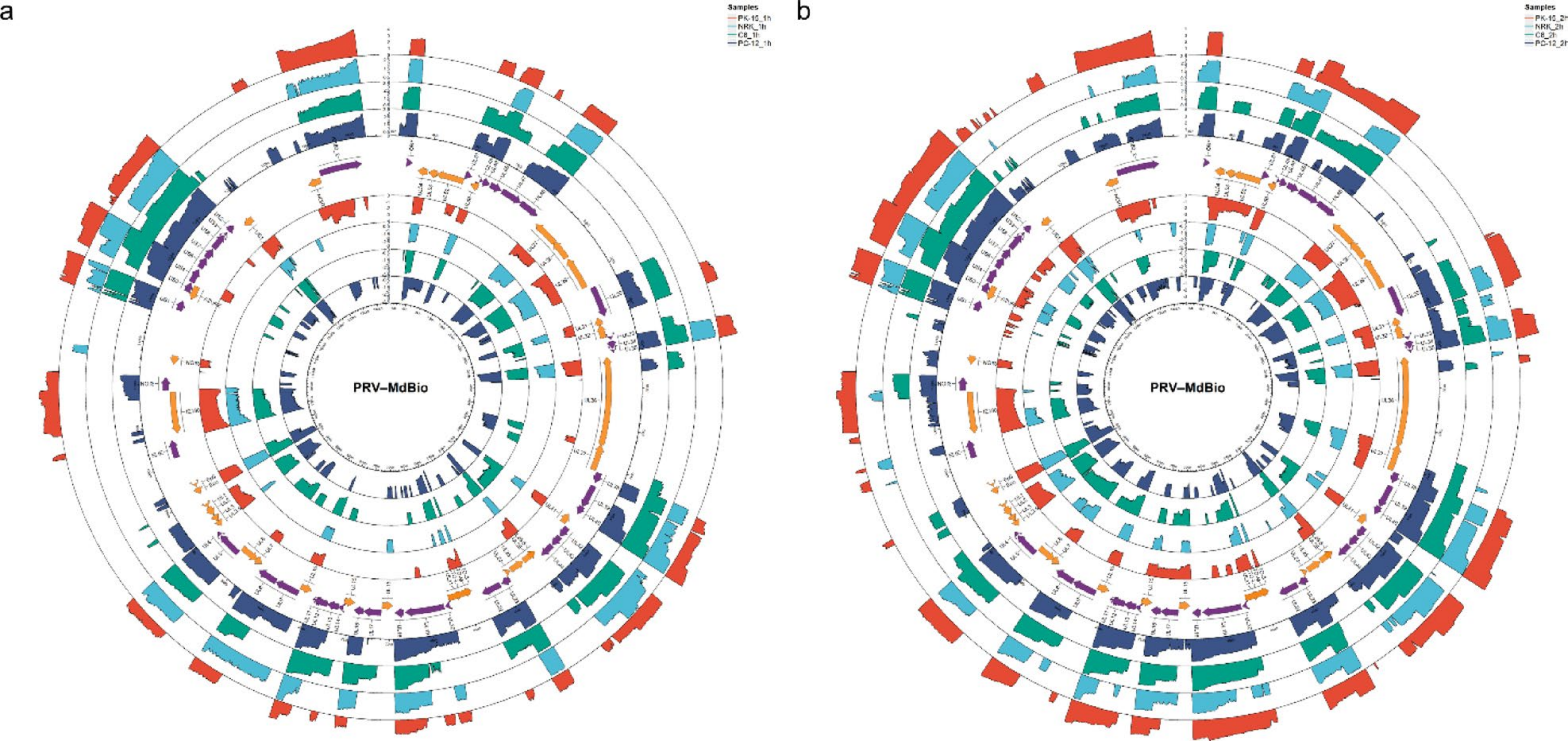
Genome-wide comparison of PRV transcript coverage across cell lines at 1-and 2-h post-infection. Circular plots display strand-specific PRV coverage along the viral genome at (**a**) 1 hpi and (**b**) 2 hpi. Coverage is shown as log₁₀(read count + 1). Each ring represents one host cell line (PK-15, NRK, PC-12, C6), arranged concentrically. Y-axis scaling is applied per panel to preserve within-time-point coverage structure while avoiding bias introduced by differences in viral read proportions across cell lines. Canonical TSS positions are indicated at their genomic coordinates. Gene models are shown in the central track. Plus-strand features are plotted on the outside and minus-strand features on the inside. Ring colors correspond to cell lines as indicated in the legend.

We next examined the TSS activity across infection time and host backgrounds (Fig. 4a and c). Time-course modeling with moanin (q ≤ 0.05) identified 49 canonical TSSs with significant inter-line or inter-group differences. While Fig. 5a and b illustrate TSS and coverage profiles for the 1 and 2 hpi samples, Supplementary Figures S2–S7 display the corresponding genome-wide TSS landscapes with additional heatmaps for canonical TSS abundances at all six time points (1, 2, 4, 6, 8, and 12 hpi).

**Fig. 6:**
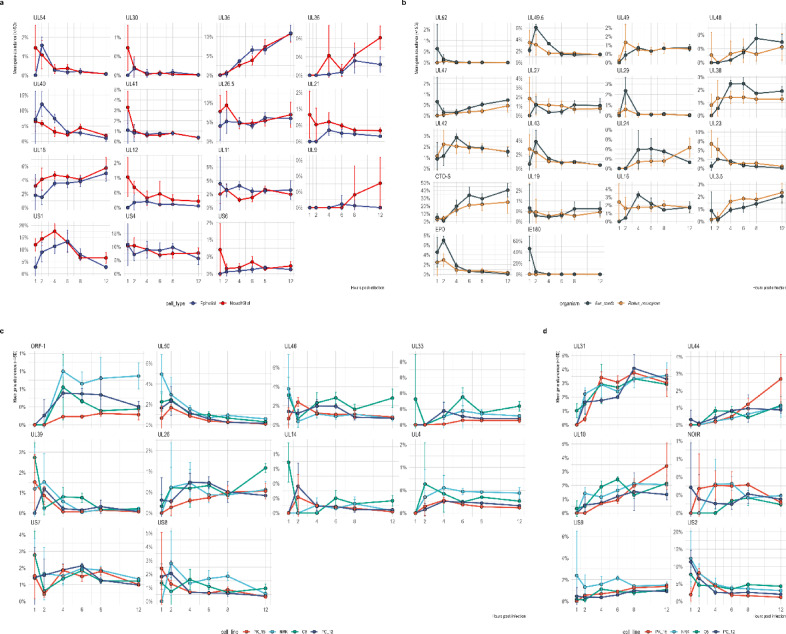
Dynamics of TSSs with significantly different temporal profiles. Line plots show mean TSS abundances (percentage of viral reads, y-axis) across the infection time-course (x-axis, hours post-infection). Each panel corresponds to genes identified as significantly differentially expressed at the TSS level in moanin time-course analyses. Colored lines denote cell lines: PK-15 (red), NRK (teal), C6 (green), and PC-12 (blue). Error bars indicate standard errors across biological replicates. (**a**) Genes significant in the cell-type model (renal/epithelial vs neural/glial; n = 15). (**b**) Genes significant in the organism model (*Sus scrofa* vs *Rattus norvegicus*; n = 18). (**c**) Genes significant in the cell-line model (individual cell-line contrasts; n = 10). (**d**) Genes significant in all three additive models (cell line, host species, and cell type; n = 6).

Grouping the cell lines by tissue origin (epithelial PK-15 and NRK versus neural/glial C6 and PC-12) revealed 21 significant TSS differences in the time-course model (q ≤ 0.05; Fig. 6a). These differences did not show a strong unidirectional bias. Neural and glial cells displayed elevated cumulative output at several loci, most prominently the regulatory gene *us1* and the capsid-associated *ul18*, with additional increases at *ul12* and *ul21*. In contrast, epithelial lines showed greater activity at loci including *ul40*, *ul11*, and *ul35*. Thus, tissue-origin effects are locus-specific and involve both regulatory and structural genes rather than forming a strictly functional dichotomy.

The organism-level contrast (*Sus scrofa* versus *Rattus norvegicus*) identified 24 significant gene TSSs in the time-course model (Fig. 6b). Porcine cells exhibited higher cumulative usage at loci including *cto-s, ie180*, and *ul38*, whereas rat-derived lines showed significantly stronger activation of *us2* and *ul23*. These findings indicate that species-level effects are present but not uniformly directional across the viral genome.

Pairwise cell-line comparisons further resolved these relationships. PK-15 versus C6 exhibited the largest number of significant time-course differences (34 genes), followed by PK-15 versus NRK (26) and PK-15 versus PC-12 (22), corresponding primarily to the cell-line model (Fig. 6c) and shared intersections across all three models (Fig. 6d). Timepoint-resolved tests indicated prominent differences at 1–2 hpi and again at 12 hpi, with fewer significant differences detected at intermediate time points.

Cumulative ΔAUC analysis supported the moanin results and highlighted TSS groups with pronounced host-dependent differences (Supplementary Fig. S8). In the PK-15 – C6 comparison, TSSs associated with regulatory, replication-linked, or structural functions—including *cto-s*, *ie180*, *ul49.5*, *ul38*, and *ul44*—showed higher cumulative TSS output in PK-15. Conversely, *ul26.5*, *ul18*, *ul23*, *us2*, *us4*, and *ul40* exhibited increased cumulative activity in C6. The PK-15 – NRK comparison showed a similar pattern: PK-15 preferentially activated *cto-s*, *ie180*, *ul49.5*, *ul38*, and *ul44*, whereas NRK exhibited stronger TSS usage at *us2*, *us4*, *ul18*, *ul23*, and ORF-1. PK-15 – PC-12 differences followed the same general structure but were milder overall. PK-15 displayed higher cumulative activity at *cto-s*, *ie180*, *ul38*, *ul44*, and *ul49.5*, while PC-12 showed stronger cumulative activation at *us2*, *ul18*, *ul23*, and loci within the *ul33–ul35* region and *ul4*. Supplementary Tables S6–S8 provide the full model statistics (S6), mean abundances across time points and cell lines (S7), and the corresponding ΔAUC values (S8).

Despite variation in magnitude, the directional structure of TSS activity remained consistent across comparisons. PK-15 preferentially amplified initiation at the major transcriptional regulator *ie180*, the OriL-associated *cto-s* transcript, and multiple capsid, tegument, and envelope loci (including *ul38*, *ul44*, and *ul49.5*), indicating enhanced regulatory drive and structural output. In contrast, the rodent lines—including both epithelial NRK and the neural/glial C6 and PC-12 cells—showed proportionally greater activation of selected immune-modulatory genes (*us2*, *us4*) together with specific capsid and nucleotide-metabolism loci (e.g., *ul18*, *ul26.5*, *ul23*), reflecting a redistribution of structural and host-interaction modules rather than a simple immune bias. These quantitative differences are evident in the temporal trajectories of significant loci (Fig. 6a–d) and in cumulative output comparisons (Supplementary Fig. S8). Importantly, the temporal cascade itself remained intact across hosts; the differences represent modulation of transcriptional amplitude rather than reordering of kinetic classes. The pronounced amplification of *ie180* in PK-15 and the relative enhancement of *us1*—most clearly in C6—mirror this genome-wide rebalancing, linking early regulatory strength to downstream structural and host–associated transcriptional output.

### TES usage varies between hosts across gene clusters

We next investigated transcription end site (TES) activity to characterize differences in transcript termination and cumulative 3′-end usage across host backgrounds. Genome-wide TES coverage profiles at each infection time point (1–12 hpi) are shown in Supplementary Figures S11–S16, with coterminal cluster abundance heatmaps displayed alongside coverage tracks. TES kinetics followed the canonical temporal cascade across all cell lines: *ie180* peaked early, *ep0* peaked early and then declined, *us1* peaked at mid-early times (4–6 hpi), and structural coterminal clusters such as *ul48–47–46*, *ul42–43–44*, *ul33–34–35*, and *cto* accumulated predominantly at 6–12 hpi (Supplementary Fig. S11–S16). These kinetic relationships were conserved across hosts, although the magnitude of TES output varied between cell lines.

Time-course modeling with moanin (q ≤ 0.05) identified 29 significant TES-associated gene clusters in the cell-line model. The temporal expression trajectories of significantly different TES clusters are shown in Supplementary Fig. S10a–d. Among the pairwise contrasts, PK-15 versus NRK showed the strongest divergence (27 significant clusters), followed by PK-15 versus C6 (25) and PK-15 versus PC-12 (19). Several TES clusters were significant across all three PK-15–based comparisons, including *ie180*, *ul29–28–27*, *ul33–34–35*, *ul42–43–44*, *ul48–47–46*, *ul51–ul49.5–49*, *ul10*, *ul20–19–18*, *ul23–22*, *ul24–25–26–26.5*, and *cto*. The *ep0* and the *us8–9–2* were frequently significant but not uniformly across all three contrasts. These results indicate that a shared set of structural, envelope-associated, and regulatory TES clusters is broadly sensitive to host-cell background (Supplementary Fig. S10a–d).

At the group level, the organism model (*Sus scrofa* versus *Rattus norvegicus*) detected 24 significant TES clusters with mixed directionality (Supplementary Fig. S10c). Clusters with enhanced TES usage in the porcine background included *ul33–34–35*, *ul42–43–44*, *ul48–47–46*, *ul10*, *ul51–ul49.5–49*, and *cto*. In contrast, the rodent background showed increased TES activity for *us8–9–2*, ORF-1, *ul20–19–18*, and *ul23–22*. The cell-type model (renal/epithelial versus neural/glial) yielded 10 significant TES clusters, also with mixed directionality (Supplementary Fig. S10d), indicating that host species exerts a stronger influence on TES usage than tissue identity in this dataset.

Cumulative ΔAUC analysis supported the moanin results and highlighted TES clusters with pronounced host-dependent differences (Supplementary Fig. S9). Structural and replication-adjacent regions—particularly *ul48–47–46*, *ul42–43–44*, *ul51–ul49.5–49*, and *cto*—showed consistently higher cumulative TES output in PK-15 than in the rodent lines (Supplementary Fig. S9). *ul10* and *ul33–34–35* generally followed this pattern but displayed contrast-specific exceptions: *ul10* showed slightly reduced cumulative TES output in PK-15 relative to C6, and *ul33–34–35* showed reduced cumulative output in PK-15 relative to PC-12 (Supplementary Fig. S9).

In contrast, several TES clusters displayed enhanced cumulative activity in rodent backgrounds (Supplementary Fig. S9). The strongest increases were observed for *us8–9–2*, followed by ORF-1, *ul20–19–18*, and *ul23–22*. Some clusters exhibited heterogeneous behavior across comparisons: *ul24–25–26–26.5* showed reduced TES usage in PK-15 relative to C6 and NRK but slightly higher cumulative output than in PC-12. Similarly, *ul38–39–40* consistently showed lower cumulative TES output in PK-15 compared with all three rodent lines and was also significant in the cell-type model (Supplementary Fig. S10d), indicating a modest but reproducible tissue-associated effect.

Overall, TES-based analysis revealed a coherent structure of host-dependent quantitative differences analogous to that observed at the TSS level. Across all hosts, the temporal hierarchy of termination events was preserved (Supplementary Fig. S11–S16), but the cumulative balance of 3′-end usage differed systematically (Supplementary Fig. S9): PK-15 preferentially utilized multiple structural and replication-linked TES clusters, whereas rodent lines showed elevated termination at several immune- and envelope-associated clusters. These differences were prominent at early time points (1–2 hpi) and again at late infection, peaking at 12 hpi (Supplementary Fig. S15–S16), while maintaining the conserved temporal ordering of gene activation across cell lines. Supplementary Tables S9–S11 provide the full TES-level model statistics (S9), mean TES abundances across time points and cell lines (S10), and the corresponding cumulative ΔAUC values for pairwise comparisons (S11).

### Transcript class composition remains broadly stable across hosts and time

Classification of PRV transcripts revealed a highly consistent class structure across infection stages and host backgrounds (Supplementary Fig. S17a). Canonical mRNAs constituted the dominant transcript class already at 1 hpi in all four cell lines, accounting for approximately 67–68% of viral transcripts, and increased further toward late infection. This increase was most pronounced in PK-15 and PC-12 cells, where canonical usage rose to ~ 84% and ~ 83% by 12 hpi, respectively, while more moderate gains were observed in NRK and C6 cells. Putative and truncated transcript classes declined gradually over the infection course in all hosts, decreasing by ~ 5–10 percentage points between 1 and 12 hpi (Supplementary Fig. S17b). Polygenic transcripts were most abundant early in infection, particularly in the rodent-derived lines, but consistently diminished toward late time points, contributing only a minor fraction of total viral reads by 12 hpi. Non-coding RNAs remained rare throughout infection, accounting for well below 1% of transcripts in all conditions. Despite measurable host- and time-dependent shifts, transcript class composition showed limited dynamic range compared with other regulatory layers. Differences between cell lines were primarily reflected in the magnitude of canonical enrichment rather than in the emergence of distinct class profiles, indicating that host-specific and temporal variation in PRV transcription is captured more effectively at the isoform, transcription start site, and termination site levels than by transcript class composition alone.

### DTU analysis reveals host-associated and time-driven isoform shifts

To evaluate host- and time-dependent differences in viral isoform usage, we applied a DRIMSeq–stageR differential transcript-usage (DTU) workflow across four additive models. The cell-line model contrasted each rodent line (NRK, PC-12, C6) with the porcine PK-15 reference while adjusting for infection time; the cell-type and organism models generalized these contrasts to epithelial versus neural/glial backgrounds and to porcine versus rodent species, respectively. The time model evaluated infection-stage–dependent differences across all pairwise contrasts among six timepoints (1, 2, 4, 6, 8, and 12 hpi; 15 contrasts in total). A stage-wise FDR threshold of 0.05 and an absolute ΔPSI cutoff of 0.05 were applied throughout.

#### Cell-line–resolved DTU

**Fig. 7:**
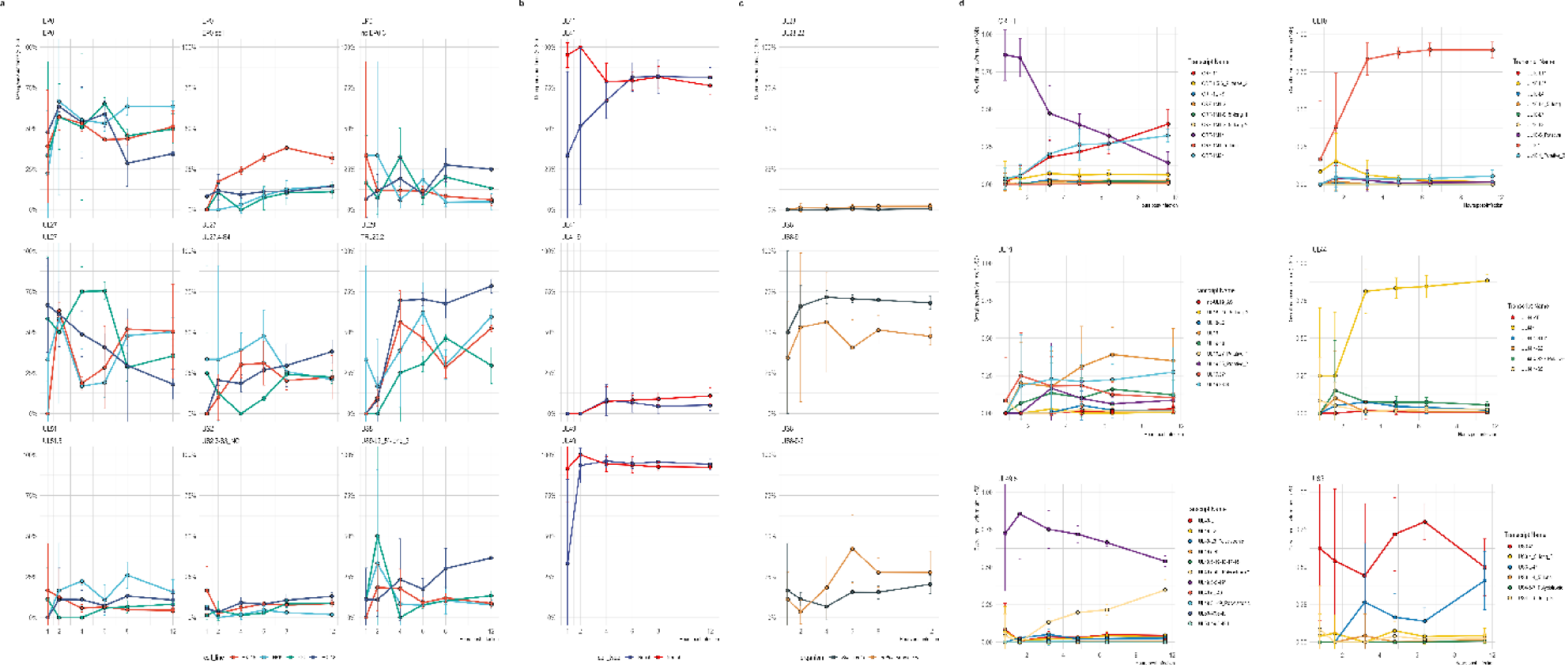
Host- and time-associated differential transcript usage (DTU) in PRV infection. (**a**) Representative isoform trajectories from the cell-line additive model, illustrating differential usage across PK-15, NRK, C6, and PC-12 cells. (**b**) Cell-type additive model (epithelial vs neural/glial), highlighting modest but reproducible isoform shifts at selected loci. (**c**) Organism additive model (porcine vs rodent), demonstrating species-dependent modulation of *us8–us9* and *ul23–ul22* regions. (**d**) Time model across six infection stages (1–12 hpi), showing extensive temporal remodeling, including near-complete isoform switching at *ul10* and progressive compaction at *ul49.5* and *us3* loci. Lines represent mean normalized transcript proportions; error bars indicate standard deviation across biological replicates.

The cell-line additive model identified 84 DTU events across 24 genes, indicating substantial isoform divergence between individual host backgrounds. DTU events were concentrated at a limited number of multivalent loci, most prominently *ep0*, the *ul29–ul27* replication cluster, and the *us8–us9* region (Fig. 7a). Representative examples included alternative EP0 isoforms (EP0, EP0-sp1, nc-EP0-3), replication-associated transcripts at the *ul29* locus and isoforms of the structural/late gene *ul27*. Additional shifts were observed in *ul51* and *us9*, highlighting fine-scale modulation across multiple functional classes. Pairwise decomposition showed that PK-15 versus NRK and PK-15 versus C6 together accounted for the majority of DTU events, whereas the PK-15 versus PC-12 comparison contributed fewer, indicating that species background exerts a stronger influence on isoform structure than differences between neural and glial lineages.

#### Cell-type–resolved DTU

The epithelial-versus–neural/glial comparison yielded 8 DTU events across 6 genes, confirming that tissue background introduces moderate but detectable isoform remodeling (Fig. 7b). In this model, significant shifts were observed at *ul41* and *ul49* loci. ΔPSI values were comparatively modest, indicating that tissue identity exerts a subtler influence on isoform architecture than either species background or infection stage.

#### Species-associated DTU

Comparisons between porcine and rodent species identified 15 DTU events across 11 genes. Species-associated DTU was most prominent within the *us8–us9* region, with US8-9 and US8-9–2 isoforms displaying clear species-dependent shifts, while in the *ul23–ul22* locus UL23-22 further illustrated coordinated modulation of adjacent genomic regions. These shifts parallel TSS- and termination-level differences detected at these loci and highlight coordinated quantitative reshaping of isoform output across species.

#### Temporal DTU dominates the isoform landscape

Infection stage produced the most extensive DTU response, identifying 352 DTU events across 20 genes across 15 pairwise time contrasts (Fig. 7d). Because multiple contrasts were evaluated, individual transcripts frequently contributed to several DTU calls, and ΔPSI values reflect the largest observed magnitude. Temporal DTU primarily reflected progressive compaction of long or polygenic isoforms during later infection stages. The most dramatic remodeling occurred at *ul10*, where the long isoform (UL10-L3) exhibited ΔPSI values approaching − 0.98 in early-versus-late comparisons. A similarly pronounced, though less extreme, pattern was observed at orf-1, where early-versus-late contrasts yielded ΔPSI values reaching approximately − 0.65, consistent with substantial reduction of the longer isoform during late infection (Supplementary Fig. S18C). Strong temporal shifts were also detected at ul49.5, us3, orf-1, ul19, and ul44 loci, indicating widespread restructuring of transcript architecture as infection progressed.

#### Cross-model structure of DTU

Most DTU-positive genes appeared in more than one host-associated model, particularly *ep0* and US8–US9, underscoring their central roles in isoform-level plasticity. However, the time model remained the most distinct, exhibiting both the highest number of events and the largest ΔPSI magnitudes. Collectively, these results indicate that PRV isoform architecture is shaped by a hierarchical structure of determinants: dominant temporal remodeling, superimposed species-level divergence, and finer cell-line–specific tuning. The full DRIMSeq–stageR results, including per-transcript statistics for all four models and all evaluated contrasts (time-course and pairwise timepoint comparisons), are provided in Supplementary Table S12.

**Fig. 8:**
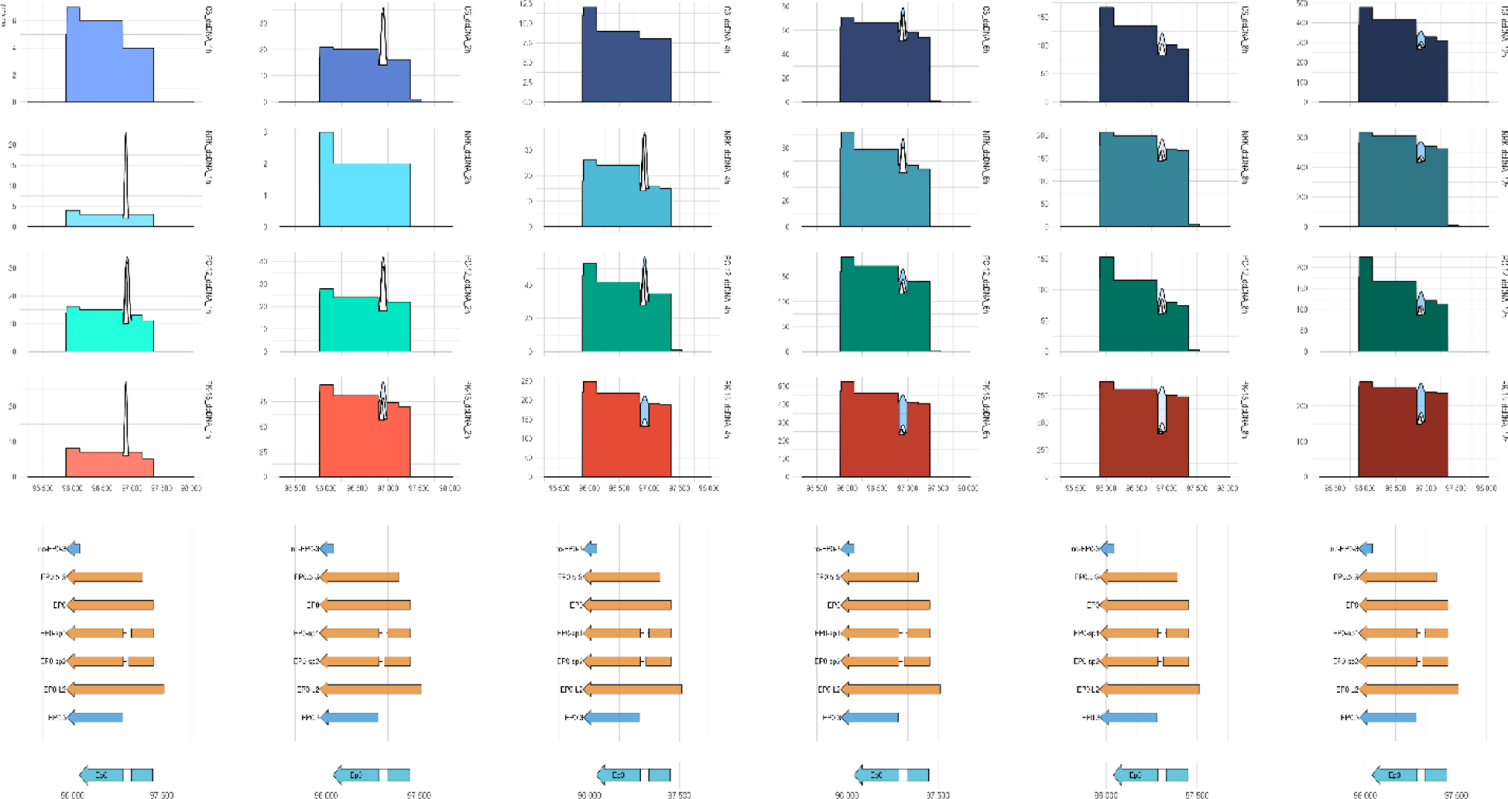
Isoform-resolved coverage and splice junction analysis of the *ep0* locus. Coverage and sashimi plots of the *ep0* genomic region across all infection stages (rows) and cell lines (columns). Splice junction arcs indicate intron usage; transcript annotations are shown below. The *ep0* gene exhibits two introns and multiple isoforms, including EP0-sp1 and nc-EP0-3. Quantitative differences in splice junction usage and transcript balance are observed across host backgrounds and over time, consistent with *ep0*’s recurrent identification in host-associated DTU models.

The most frequently appearing transcripts across the models were those of the *ep0* gene, including its alternative splice forms and non-coding variants. They appeared repeatedly across host-associated models, but not in the time model. Figure 8 shows the coverage and splicing architecture of the *ep0* locus. The strongest effect and the lowest p-value (stage-wise adjusted *p*-value < 5 × 10⁻5) were observed in the cell-line model. Indeed, the EP0-sp1 splice isoform displayed a mean PSI of 0.239 in PK-15 cells, compared with 0.110 in PC-12 and 0.065 in both NRK and C6. In contrast, the EP0.3 isoform showed lower representation in PK-15 (0.070) but was more abundant in rodent-derived lines, reaching 0.225 in C6, 0.171 in PC-12, and 0.119 in NRK. The canonical EP0 transcript remained dominant across all backgrounds (PSI ~ 0.48–0.54) with only modest intercellular variation.

## Discussion

By integrating direct cDNA sequencing, direct RNA sequencing, and CAGE-seq across multiple host backgrounds, this study substantially extends previous PRV transcriptome surveys performed in a single porcine cell line^10,11,33^. Canonical monogenic mRNAs remained the dominant class throughout infection and increased strongly at late stages in all cell lines as structural gene expression intensified. The transcriptome also contained extensive 5′ variation, polygenic readthrough RNAs, and putative 5′-truncated forms, whereas antisense, splice-derived, and complex RNAs were comparatively rare, consistent with related alphaherpesviruses such as VZV, BoHV-1, and EHV-1^7–9^. The newly detected CEP transcripts were reproducibly present across cell lines, although present in low abundance, supporting their status as *bona fide* PRV RNAs. Together with long 5′ UTR and polygenic isoforms, these transcripts indicate that TSS variability and controlled readthrough are major drivers of transcriptional diversity, rather than large-scale splicing or structural rearrangements^14,27,33,35^.

Our experimental design using one porcine epithelial (PK-15) and three rodent lines (epithelial NRK, glial C6, neuron-like PC-12) enabled complementary dissection of species and cell-type effects: species-level effects were assessed using a porcine-versus-rodent model, while tissue-type differences were evaluated using an epithelial-versus–neural/glial contrast; additional PK-15–centered comparisons provided cell-line–resolved resolution of host-associated variation. The use of a single porcine cell line represents a limitation, as some apparent species-specific differences could potentially reflect PK-15-specific characteristics. However, well-characterized porcine glioma or neuron-like cell lines directly comparable to C6 and PC-12 are not currently available. Although a porcine olfactory bulb neuroblast line (OBGF400) has been described^36^, it has not been widely adopted in PRV research and lacks the extensive virological and transcriptional characterization available for PK-15, C6, and PC-12, limiting its suitability for controlled cross-species comparisons. Importantly, several observations support that our findings reflect genuine host biology. The three rodent lines exhibited broadly consistent patterns—particularly elevated immune- and envelope-associated gene expression relative to PK-15—despite distinct tissue origins. Key species-dependent differences, such as dramatically higher *ie180* expression in PK-15, align with known biological differences in PRV pathogenesis between porcine and rodent hosts. In our dataset, the rat epithelial NRK line consistently displayed an intermediate transcriptional phenotype between porcine PK-15 and rodent neural/glial lines, a pattern compatible with reported host- and epithelial cell-type–dependent differences in interferon-mediated restriction and PRV countermeasures^23–25^.

Although viral transcript abundance differed substantially across host species and cell types, the structural composition of the PRV transcriptome remained remarkably stable. Only a small number of isoforms showed host- or cell-type–restricted detection under stringent annotation criteria, indicating that host background primarily modulates transcript output rather than transcript architecture. While it remains possible that extremely low-abundance isoforms may arise in specific cellular environments, such events were rare and did not constitute coherent structural subclasses. These findings suggest that PRV maintains a conserved transcriptional blueprint across divergent host contexts, with host-dependent effects operating predominantly at the level of transcriptional magnitude and temporal kinetics rather than isoform innovation.

The marked differences in viral transcript accumulation across cell lines (Fig. 2) are consistent with differences in replication efficiency or overall permissiveness. PK-15 cells, derived from the natural porcine host, exhibited the highest viral RNA accumulation, exceeding 50% of total reads by 12 hpi. In contrast, PC-12 reached approximately 34%, whereas NRK and C6 plateaued at ~ 19–20% at the same time point.

Higher replication efficiency in PK-15 coincided with stronger accumulation of late structural transcripts, whereas rodent-derived lines exhibited relatively greater representation of termination sites associated with envelope-, egress-, and immune-modulating viral genes. Importantly, despite these quantitative and locus-specific differences, the temporal IE → E → L cascade remained conserved across all four cell lines, indicating that the core regulatory program of PRV is maintained across host backgrounds while the relative weighting of functional gene clusters is context-dependent.

Across porcine and rodent cells, the regulatory order of *ie180* → *ep0* → *us1* was conserved, but the quantitative amplitude of each regulator—and the isoform balance of *ep0* and *us1*—varied across host backgrounds^12,15,18,19^. The *ie180* showed the strongest species dependence: PK-15 cells exhibited a prominent early burst, whereas NRK, C6, and PC-12 expressed substantially lower levels during the early phase. This pronounced amplitude difference is consistent with the essential role of *ie180* and its HSV-1 homolog ICP4 in initiating the lytic cascade^5,13,15^. The *ep0* was activated early in all backgrounds but displayed moderate host-dependent quantitative variation. Differences were particularly evident at the TES level, where epithelial and rodent lines showed distinct ordering across time points rather than a uniform species bias. *us1* exhibited the clearest cell-type dependence, with C6 and PC-12 showing the highest early activity and a modest enrichment for longer or spliced variants. Extended early time-course sampling in PC-12 confirmed that the weak *ie180* signal reflects genuinely low-amplitude activation rather than delayed onset. Together, these findings support a model in which the *ie180*–*ep0*–*us1* triad operates as a quantitatively tuned regulatory module whose output is modulated by host species and cell-type context rather than by changes in activation order^18,23^.

Genome-wide TSS analysis further showed that the canonical IE → E → L cascade is preserved, but promoter amplitudes vary substantially across hosts, similar to temporal patterns reported for HSV-1 and HCMV^27,29^. PK-15 preferentially activated regulatory, replication-adjacent, and structural TSSs, including *cto-s*, *ie180*, *ep0*, *ul38, ul44,* and *ul49.5*, whereas rodent-derived lines—particularly C6 and PC-12—showed stronger activation of numerous envelope- and immune-associated loci such as *us2, us4, ul18, ul23,* and *ul40*.

The observed association between reduced *ie180* amplitude in rodent cells and relatively enhanced activation of envelope- and immune-linked genes is consistent with differential early transcriptional control, potentially analogous to transient repression and subsequent derepression mechanisms described for HSV-1 IE proteins^16^, although direct mechanistic testing will be required to establish causality.

TES analysis revealed a similar hierarchy at the 3′ end: structural TES clusters such as *ul33–ul35, ul10, ul48–ul47–ul46,* and *ul51–ul49.5–ul49* showed consistently higher cumulative usage in PK-15, whereas TES positions linked to immune- and envelope-associated genes (*us8–us9–us2, orf-1, ul20–ul19–ul18, ul23–ul22)* were enhanced in rodent-derived lines. Comparable host- and time-dependent differences in 3′-end usage have been described in BoHV-1 and EHV-1^14,34^. Rather than indicating disruption of the canonical kinetic cascade, these patterns reflect host-dependent quantitative rebalancing of TSS and TES usage, with porcine cells showing stronger accumulation of structural and replication-associated termini and rodent cells exhibiting relatively increased termination at envelope- and host-interaction–associated loci.

Principal-component analysis and class-composition profiles further support this view. All cell lines converged toward a late-stage transcriptome dominated by canonical mRNAs, with putative and polygenic classes declining over time. Neural and glial cells retained slightly higher early proportions of putative and polygenic RNAs, but by 12 h post-infection all backgrounds were strongly canonical. The strongest temporal signal was captured at the isoform level, where PC1 values showed the highest adjusted R^2^ with infection time, followed by gene-level and TSS-based matrices, with TES-based variation contributing a weaker but consistent component. These differences underscore a key advantage of long-read sequencing: isoform-resolved quantification captures infection-stage progression and host differences more effectively than gene-level or boundary-only summaries obtainable from short-read data alone^9,27,28,30,32,35^.

Differential transcript usage analysis revealed extensive isoform remodeling shaped jointly by host background and infection stage. Host-associated contrasts identified DTU events concentrated at a limited number of loci rather than being uniformly distributed across the genome. In the cell-line model, isoform shifts were particularly evident at the *ep0* locus and within the *ul29–ul27* replication cluster, with additional contributions from *ul51* and *us9*-associated transcripts. These patterns indicate that individual cellular environments fine-tune replication- and structural-associated transcript architectures without disrupting the overall kinetic cascade.

Cell-type–associated DTU was more restricted in scope and effect size. Epithelial versus neural/glial comparisons primarily involved *ul41* and *ul49* loci, where moderate but consistent differences in transcript balance were observed. These shifts were quantitatively smaller than those seen in species or temporal contrasts, supporting the view that tissue background modulates isoform output in a subtler, secondary manner. Species-associated DTU highlighted the *us8–us9* region and the *ul23–ul22* locus as major sites of divergence between porcine and rodent hosts. These loci also displayed coordinated TSS- and TES-level differences, indicating that species-dependent regulation operates across multiple layers of transcriptional control rather than at a single structural boundary. Infection stage exerted the strongest influence on isoform architecture. Temporal remodeling was characterized by progressive reduction of long or polygenic isoforms and increasing dominance of compact transcripts at late stages. The *ul10* exhibited the most pronounced switch, with near-complete replacement of the long UL10-L3 isoform in early-versus-late comparisons. Strong time-dependent restructuring was also observed at *ul49.5, us3, orf-1, ul19,* and *ul44* loci. These widespread transitions indicate that temporal compaction of transcript architecture represents a central organizing principle of PRV transcription.

Among all loci examined, *ep0* emerged as the most consistently host-sensitive regulatory node, appearing across multiple host-associated DTU models and exhibiting complex isoform architecture involving two introns and several alternative splice and non-coding variants. Quantitative PSI analysis demonstrated a pronounced species-dependent redistribution of EP0 isoforms rather than a simple change in total transcript output. In PK-15 cells, the EP0-sp1 splice isoform reached a mean PSI of 0.239, compared with 0.110 in PC-12 and 0.065 in both NRK and C6, corresponding to ΔPSI values of up to 0.174 in the cell-line model. Conversely, the EP0.3 isoform was relatively depleted in PK-15 (0.070) but enriched in rodent-derived lines, particularly C6 (0.225). Importantly, the canonical EP0 transcript remained dominant across all backgrounds (PSI ~ 0.48–0.54), indicating that host effects operate primarily through rebalancing of splice isoforms rather than wholesale transcriptional activation or repression. The coverage and sashimi profiles in Fig. 8 further illustrate dynamic modulation of splice junction usage across hosts and timepoints. Together, these findings support a model in which EP0 may function as a dosage-sensitive regulatory hub whose isoform composition is selectively tuned by species background, providing a flexible mechanism for adjusting early transcriptional control without altering the conserved kinetic cascade.

Collectively, these results support a hierarchical model of PRV transcriptional regulation in which dominant temporal remodeling is superimposed upon species-level divergence and finer cell-line–specific tuning. Rather than altering the conserved IE → E → L cascade order, host environment reshapes transcript architecture quantitatively, adjusting isoform balance within key regulatory and structural loci.

Beyond the cell line selection discussed above, several limitations should be noted. This study used immortalized cell culture, which may not fully recapitulate in vivo infection, particularly latency and reactivation. We examined a single PRV strain (MdBio), and strain-specific differences may limit generalizability. Our six-time-point sampling captures major transitions but may miss transient events. In addition, although transcript architecture was highly conserved across cell lines under stringent annotation criteria, the absence of full-length dRNA validation in NRK cells and the possibility of extremely low-abundance, cell-specific isoforms falling below detection thresholds represent minor technical constraints of the current study. Finally, functional validation of newly identified isoforms and their biological roles remains to be determined.

In summary, across four cellular environments, PRV maintains its canonical kinetic cascade while quantitatively tuning TSS activity, TES usage, and isoform selection in a species- and tissue-dependent manner. Porcine kidney cells preferentially accumulate structural gene transcripts, whereas rodent neural and glial lines show relatively enhanced representation of envelope- and host-interaction–associated genes, with NRK exhibiting an intermediate phenotype. Previous studies demonstrating species-specific interferon restriction and cell-type–dependent modulation of PRV infection^19,23–26^ support the biological plausibility of these host-associated transcriptional differences. Isoform- and boundary-level heterogeneity enable fine-scale contextual adaptation without altering the conserved temporal backbone, with the *ie180*–*ep0*–*us1* triad functioning as a central quantitative regulatory node that integrates host- and time-dependent signals^18,19,23^. Comparable principles of promoter plasticity and isoform remodeling have been reported in VZV, HSV-1, HCMV, and BoHV-1^7,9,29,30,32,34^; however, to our knowledge, this study represents the first multi-host, isoform-resolved, time-resolved analysis of PRV transcription, providing a framework for dissecting how host environment shapes alphaherpesvirus transcriptional architecture and informing future investigations of latency, reactivation, and tissue-specific pathogenesis.

## Materials and methods

### Cells and viruses

In this study, we used the following four immortalized cell lines for the propagation of the MdBio strain of pseudorabies virus (PRV-MdBio^37^): the PK-15 porcine kidney epithelial cell line (ATCC CCL-33), the NRK rat kidney epithelial cell line (ATCC CRL-6509), the C6 cell line derived from a rat glial tumor (ATCC CCL-107), and the PC-12 cell line (ATCC CRL-1721) derived from a pheochromocytoma of the rat adrenal medulla, which has an embryonic origin from the neural crest. PK-15 and NRK cells were cultured in DMEM Low Glucose (1 g/L) with L-glutamine and sodium pyruvate (Capricorn Scientific), supplemented with 10% fetal bovine serum (FBS; Capricorn Scientific). C6 cells were grown in DMEM/Ham’s F-12 (DMEM/F-12) with L-glutamine (Capricorn Scientific), supplemented with 2.5% FBS and 15% horse serum (both from Capricorn Scientific). PC-12 cells were cultured in RPMI supplemented with 5% FBS and 10% horse serum (Capricorn Scientific). All media were supplemented with 1% penicillin–streptomycin solution, and cells were maintained at 37 °C in the presence of 5% CO₂. Virus stock solution was prepared as follows. PK-15 cells were infected at a multiplicity of infection (MOI) of 0.1 [MOI = plaque-forming units (pfu)/cell]. Viral infection was allowed to progress until complete cytopathic effect was observed, followed by three successive cycles of freezing and thawing of infected cells to release viruses. Each cell type was then infected at an MOI of 10 with PRV-MdBio to ensure synchronous infection despite differences in underlying permissiveness. Infected cells were incubated for 1 h at 37 °C, after which the virus suspension was removed and the cells were washed with phosphate-buffered saline. Fresh culture medium was added, and the cells were further incubated for 1, 2, 4, 6, 8, or 12 h. At each time point, the culture medium was discarded and the infected cells were frozen at − 80 °C until further use.

### RNA isolation

Total RNA extraction was performed using the NucleoSpin RNA II kit for short-read sequencing and the NucleoSpin RNA kit (both from Macherey–Nagel) for lrRNA-seq. The process began with cell collection by centrifugation, followed by lysis using a solution containing chaotropic ions, which inactivate RNase enzymes. Both DNA and RNA molecules were then bound to a silica membrane. To remove DNA, the samples were treated with DNase I solution supplied with the kit. Total RNA was subsequently eluted in RNase-free water. Further DNA decontamination was performed using the Ambion TURBO DNA-free Kit. RNA samples were stored at − 80 °C until use. For purification of the poly(A) + fraction of total RNA, the Poly(A) RNA Selection Kit V1.5 (Lexogen) was used.

### Direct cDNA sequencing

Non-amplified dcDNA libraries from the poly(A) + RNA fraction of the MdBio strain were prepared using ONT Direct cDNA Sequencing Kit (SQK-DCS109), following the manufacturer’s instructions. Briefly, 100 ng of poly(A) + RNA was used for first-strand cDNA synthesis with Maxima H Minus Reverse Transcriptase (Thermo Fisher Scientific) and SSP and VN primers from the kit. Residual RNA was then removed using the RNase Cocktail Enzyme Mix (Thermo Fisher Scientific). The second cDNA strand was synthesized using LongAmp Taq Master Mix (New England Biolabs). Double-stranded cDNA was end-repaired and dA-tailed using the NEBNext End Repair/dA-Tailing Module (New England Biolabs), followed by ligation of the sequencing adapters using the Quick Ligation Module (New England Biolabs).

### Direct RNA sequencing

For direct RNA sequencing, 500 ng of polyadenylated RNA per library was used. For each cell line, a pooled RNA sample was prepared by combining equal molar amounts of RNA from individual samples. Library preparation was performed using the ONT Direct RNA Sequencing Kit (SQK-RNA002), according to the manufacturer’s recommended protocol.

### Cap analysis of gene expression

CAGE-seq libraries were prepared from 5 µg total RNA using the CAGE Preparation Kit (DNAFORM, Japan) according to the manufacturer’s protocol and our previous description (Torma et al., 2023). Briefly, RNA was denatured with the kit RT primer at 65 °C, followed by first-strand cDNA synthesis with SuperScript III in the presence of a trehalose/sorbitol enhancer. The 5′ cap diols were oxidized (NaIO₄) and biotinylated (long-arm biotin hydrazine), single-stranded RNA was digested with RNase I, and cap-trapped molecules were enriched on streptavidin beads and released. We then performed 5′/3′ linker ligation, SAP and USER treatments, second-strand synthesis, and Exonuclease I digestion, with clean-ups using RNAClean XP/Ampure XP as appropriate. Libraries were barcoded, quantified (Qubit ssDNA HS), quality-checked (TapeStation), and sequenced on Illumina MiSeq (v3 150-cycle).

### Measurement of nucleic acid quality and quantity

Total RNA was quantified using the Qubit RNA BR Assay Kit (Invitrogen), and the amounts of the poly(A) + and rRNA-depleted RNA fractions were determined using the Qubit RNA HS Assay Kit (Invitrogen). A Qubit 4 fluorometer was used to measure final RNA sample concentrations. For cDNA samples and sequencing-ready libraries, concentrations were measured using the Qubit dsDNA 1 × HS Assay Kit (Invitrogen). RNA integrity (RIN) was assessed using the TapeStation 4150 system, and only samples with RIN values above 9.6 were used for dcDNA-seq and dRNA-seq library preparation.

### Sequencing data pre-processing

All sequencing libraries were processed in R 4.3^39^ and Bash. Nanopore dcDNA-seq signals were base-called with dorado^40^ using the super-accurate model and reads with a Phred score below 8 were discarded. The remaining reads were aligned to the PRV MdBio reference genome (LT934125.1) with minimap2 v2.24 in long-read spliced mode (-ax splice -Y -C5 -cs), preserving complete CIGAR and cs tags^41^.

In this study, we used the LoRTIA software package^42^ to detect and annotate transcripts and transcript isoforms, as described previously^35^. Sequencing adapters and homopolymer A stretches were used to identify transcription start sites (TSSs) and transcription end sites (TESs). Candidate TSSs and TESs were tested against a Poisson distribution with Bonferroni correction to remove spurious sites arising from RNA degradation, incomplete reverse transcription/PCR, or template switching. Candidate introns were accepted if they used one of the three most frequent splice consensus motifs (GT/AG, GC/AG, AT/AC) and had an abundance of at least 1‰ of the local coverage. TSSs and TESs were retained if detected by at least two different sequencing methods, and introns were considered real if present in both dRNA-seq and at least one dcDNA-seq dataset and were shorter than 10 kb. We used a relatively low abundance threshold because splice-variant frequencies can differ between cell types. Accepted TSSs, TESs, and introns were assembled into transcript models with the Transcript_Annotator module of LoRTIA. Reads were considered bona fide transcripts if observed in at least three different samples; rare very long isoforms missed by LoRTIA were added manually when they clearly extended existing models.

Transcripts were then categorized as follows: the most abundant single-ORF isoform was defined as the canonical monogenic transcript; isoforms with longer or shorter 5′ or 3′ UTRs were termed TSS or TES variants; alternatively spliced transcripts were classified as splice isoforms; transcripts with a 5′-truncated in-frame ORF were termed putative mRNAs; those with multiple tandem non-overlapping ORFs were considered polygenic; transcripts containing at least one ORF in the opposite orientation were classified as complex; and transcripts lacking ORFs or carrying ORFs < 30 nt were considered noncoding, except for short upstream ORFs (uORFs) located 5′ to a canonical ORF. Illumina CAGE-seq reads were adapter-trimmed with TrimGalore! 0.6.7^43^ and mapped with STAR 2.7.3a (SA index = 8)^44^. All BAM files were loaded via Rsamtools^45^, then parsed with data.table ^46^ to extract exon–intron structures together with the 5′- and 3′-most aligned bases of every read.

### Downstream bioinformatics analysis

Transcription-start-site discovery relied on TSSr 1.3^47^. Filtered CAGE-seq counts were reshaped into a position-by-sample matrix and supplied to TSSr as TSStable input alongside the custom BSgenome.PRV.MdBio.1.0 package. Principal-component analysis confirmed replicate agreement before replicates were merged. Tag clusters were called with the peakclu algorithm (40-nt peak distance, 10-nt extension, local-threshold 0.05, cluster-threshold 2); consensus clusters were merged at 25 nt, annotated to genes within a − 500/ + 200 nt window and written to disk for counting. For 3′-end mapping, the right-most aligned base of each dcDNA-seq read was treated as a putative TES. These coordinates were clustered in TSSr with the same parameters, but on the antisense strand. Resulting TES clusters were welded to the TSS catalogue and to reference coding regions by exact chromosome, strand and coordinate overlap, yielding a unified list of dominant TSS/TES pairs for every PRV ORF.

Nanopore alignments were processed by LoRTIA 0.9.9 for adapter-trimming, Poisson-filtered TSS/TES validation and transcript reconstruction^41^. A start or end site was retained if it appeared in at least three biological samples or in two orthogonal techniques (dcDNA-seq plus dRNA-seq or CAGE-seq) and lacked poly-dA artifacts. Splice junctions bearing GT/AG, GC/AG or AT/AC motifs, shorter than 10 kb and representing more than 0.1% of local coverage in both dRNA-seq and at least one dcDNA-seq library, were accepted. The resulting isoforms were categorized as canonical monogenic, TSS/TES variants, splice variants, 5′-truncated putative mRNAs, polygenic, complex or non-coding/uORF transcripts. Read counts whose 5′ ends fell inside an accepted TSS cluster and whose 3′ ends coincided with the canonical TES were aggregated with GenomicRanges^48^ and tidygenomics^49^, producing TSS-based and TES-based matrices covering six time-points (1–12 h), four cell lines and three replicates.

Differential time-course gene expression was assessed with moanin (voom-limma back-end)^50^. TMM-normalized counts were fitted to spline-interaction models of the form ~ cell line ns(Time, 4) (with analogous cell-type and organism terms). Empirical-Bayes-moderated statistics were corrected by Benjamini–Hochberg; genes with q < 0.05 were deemed significant. Differential transcript usage was tested with DRIMSeq^51^ 1.24 followed by stageR 1.12^52^. Isoforms contributing fewer than ten reads in three samples were filtered out. The same spline-interaction design was fitted in DRIMSeq; likelihood ratios and precision estimates were passed to stageR, whose two-stage procedure controlled the gene-level FDR at 5%.

Coverage tracks and sashimi plots were produced using ggplot2^53^ and gggenes^54^, splicejam^55^, circlize^56^ and the Rlyeh collection of R scripts** (**https://github.com/Balays/Rlyeh**).**

## Supplementary Information

Below is the link to the electronic supplementary material.

Supplementary Material 1

Supplementary Material 2

Supplementary Material 3

Supplementary Material 4

Supplementary Material 5

Supplementary Material 6

Supplementary Material 7

Supplementary Material 8

Supplementary Material 9

Supplementary Material 10

Supplementary Material 11

Supplementary Material 12

Supplementary Material 13

Supplementary Material 14

Supplementary Material 15

Supplementary Material 16

Supplementary Material 17

Supplementary Material 18

Supplementary Material 19

Supplementary Material 20

Supplementary Material 21

Supplementary Material 22

Supplementary Material 23

Supplementary Material 24

Supplementary Material 25

Supplementary Material 26

Supplementary Material 27

## Data Availability

The *fastq* files from all sequencing were uploaded to European Nucleotide Archive (ENA), under the project id PRJEB60055.

## Code availability

All R scripts that were used for the downstream analysis are available at the GitHub repository: https://github.com/Balays/VALAR-PRV-MDBIO-4cell
