# Supplementary figures and images for: Multi-platform profiling reveals host- and cell -type-specific pseudorabies virus gene expression
Source: Sci Rep. 2026 Apr 1;16:15297. doi: 10.1038/s41598-026-45990-4 (PMC13181115; doi:10.1038/s41598-026-45990-4)

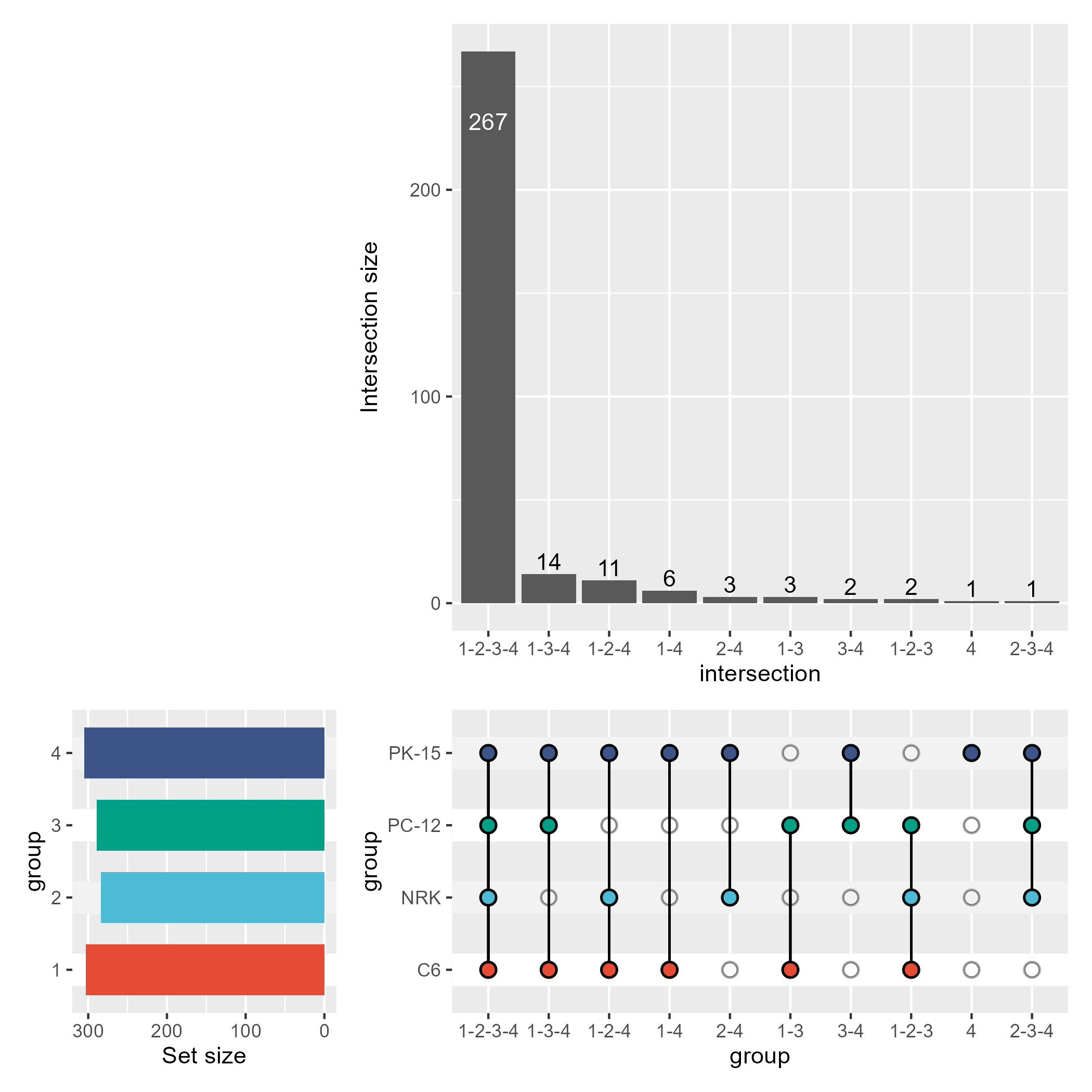

Supplement: Supplementary file 1 — Supplementary Material 1 [file 41598_2026_45990_MOESM1_ESM.png]

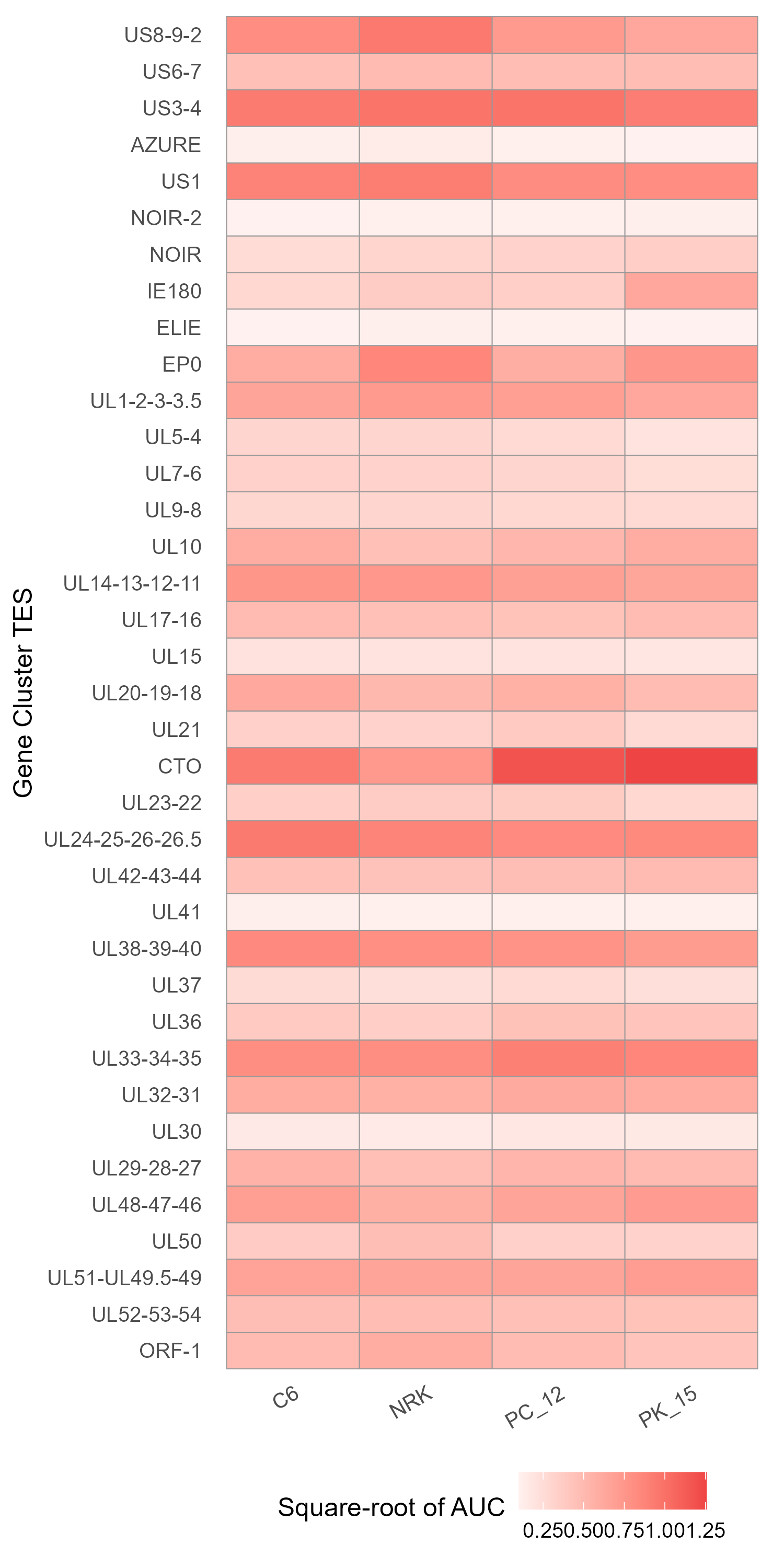

Supplement: Supplementary file 2 — Supplementary Material 2 [file 41598_2026_45990_MOESM2_ESM.png]

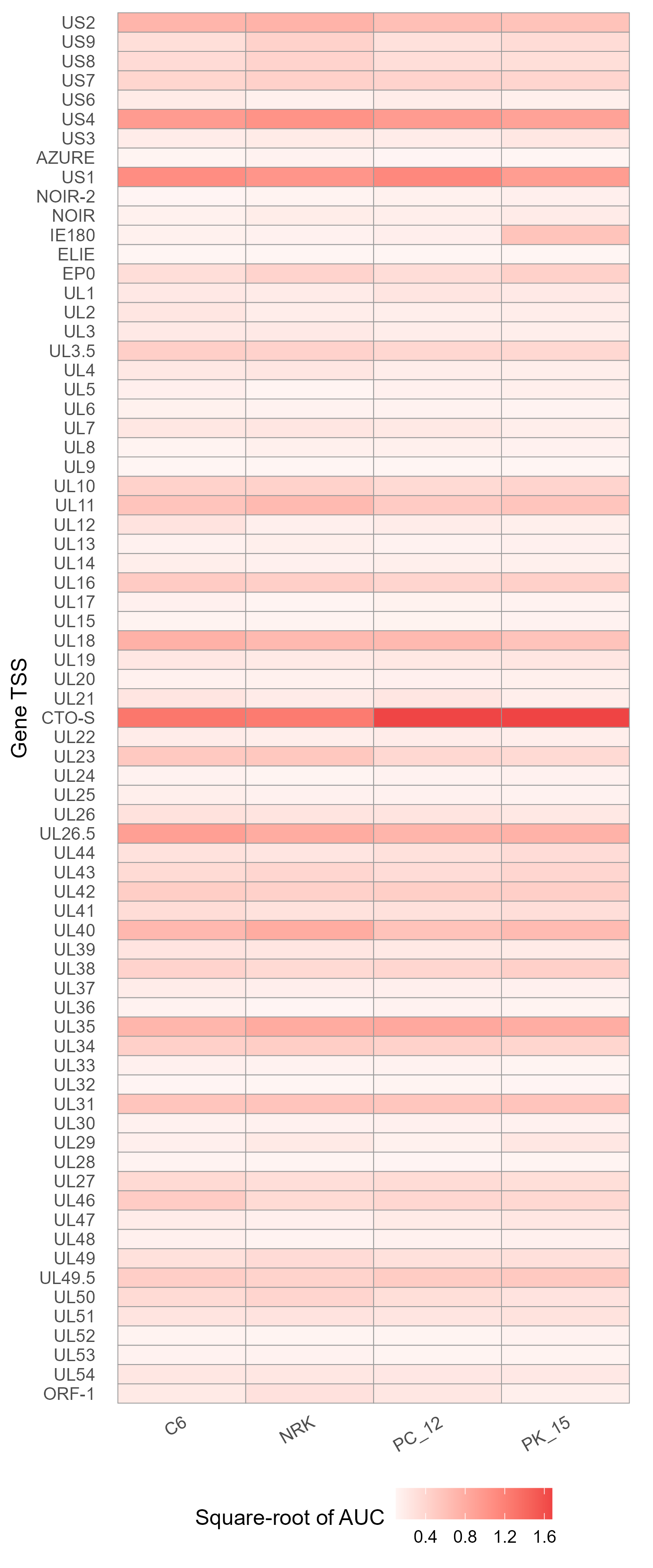

Supplement: Supplementary file 3 — Supplementary Material 3 [file 41598_2026_45990_MOESM3_ESM.png]

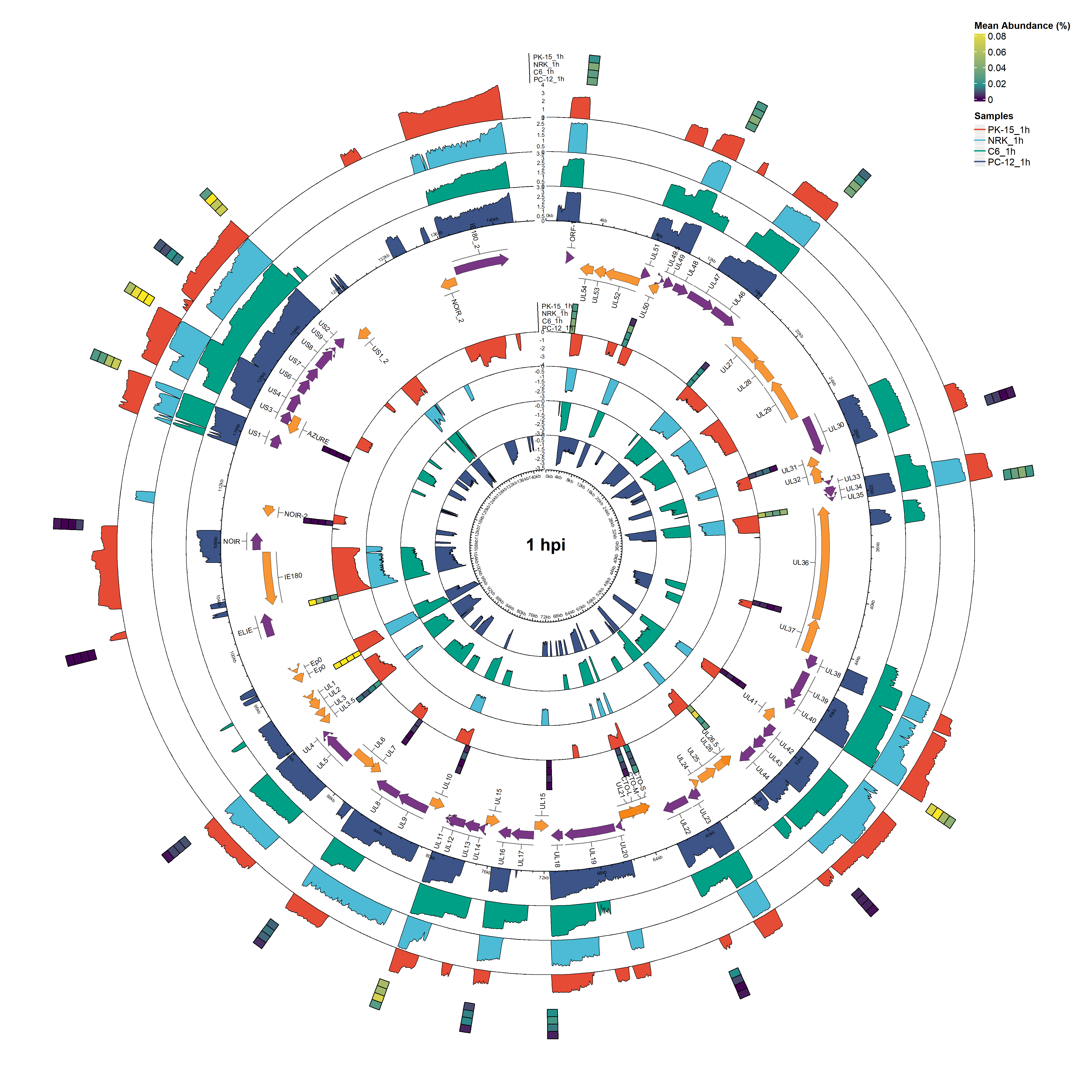

Supplement: Supplementary file 4 — Supplementary Material 4 [file 41598_2026_45990_MOESM4_ESM.png]

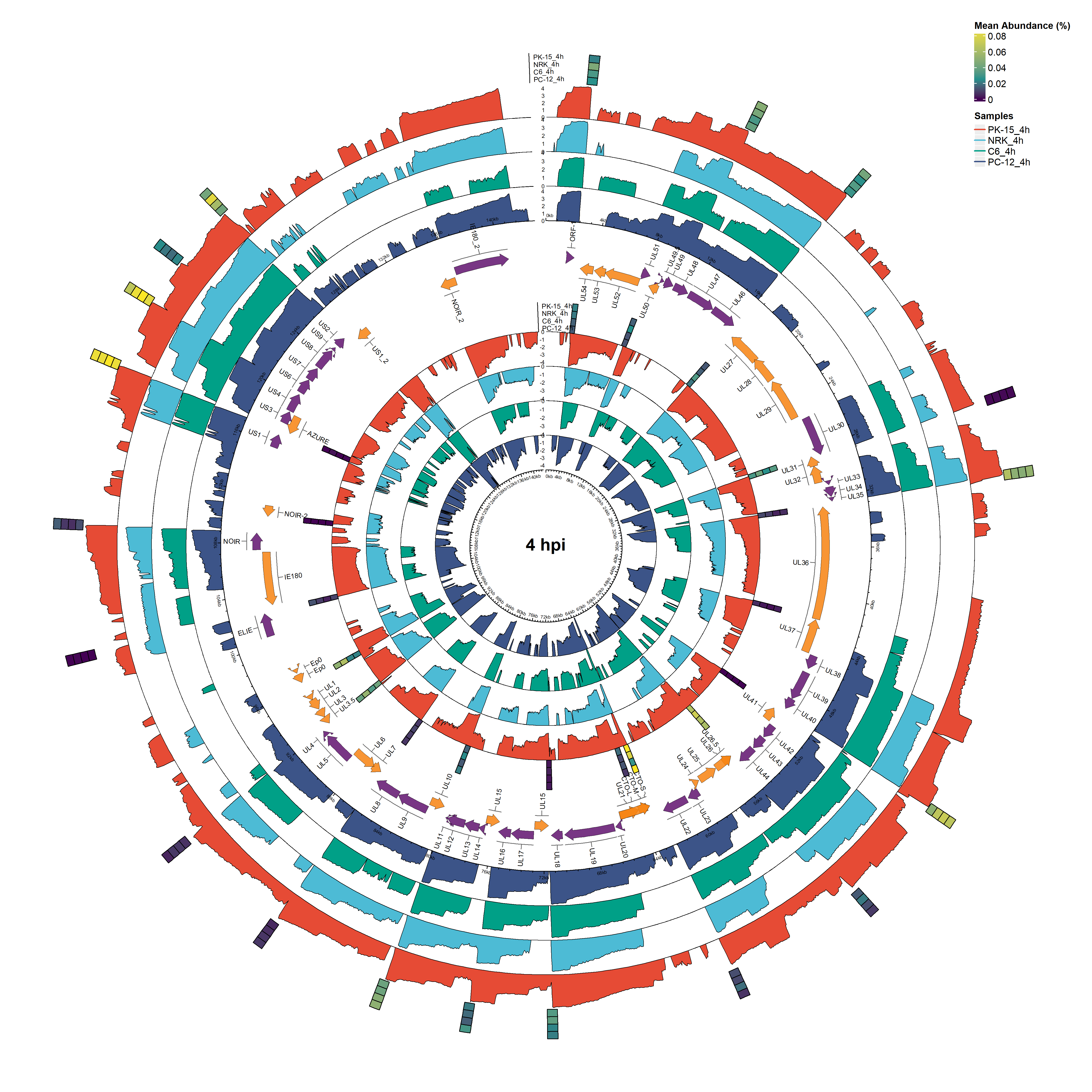

Supplement: Supplementary file 5 — Supplementary Material 5 [file 41598_2026_45990_MOESM5_ESM.png]

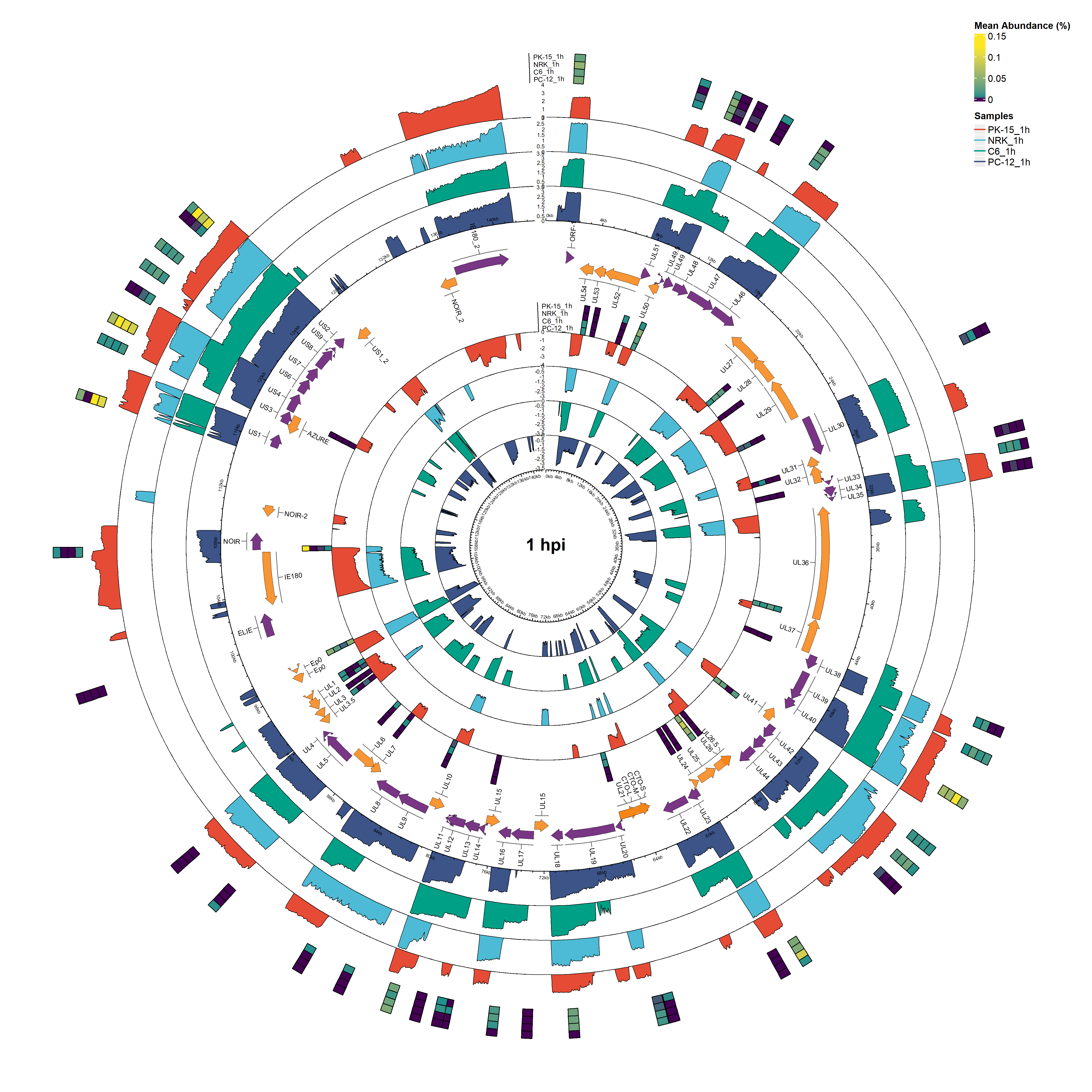

Supplement: Supplementary file 6 — Supplementary Material 6 [file 41598_2026_45990_MOESM6_ESM.png]

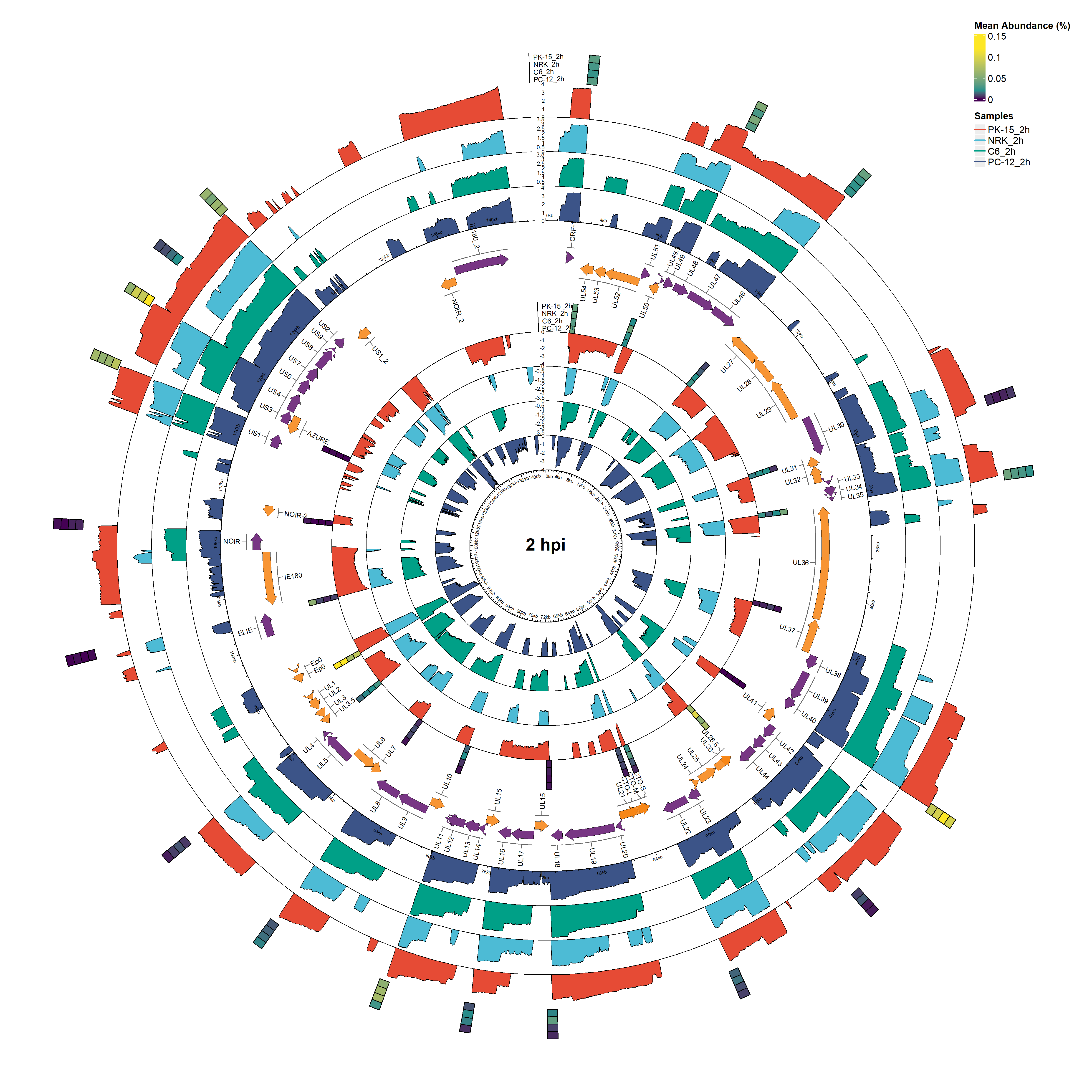

Supplement: Supplementary file 7 — Supplementary Material 7 [file 41598_2026_45990_MOESM7_ESM.png]

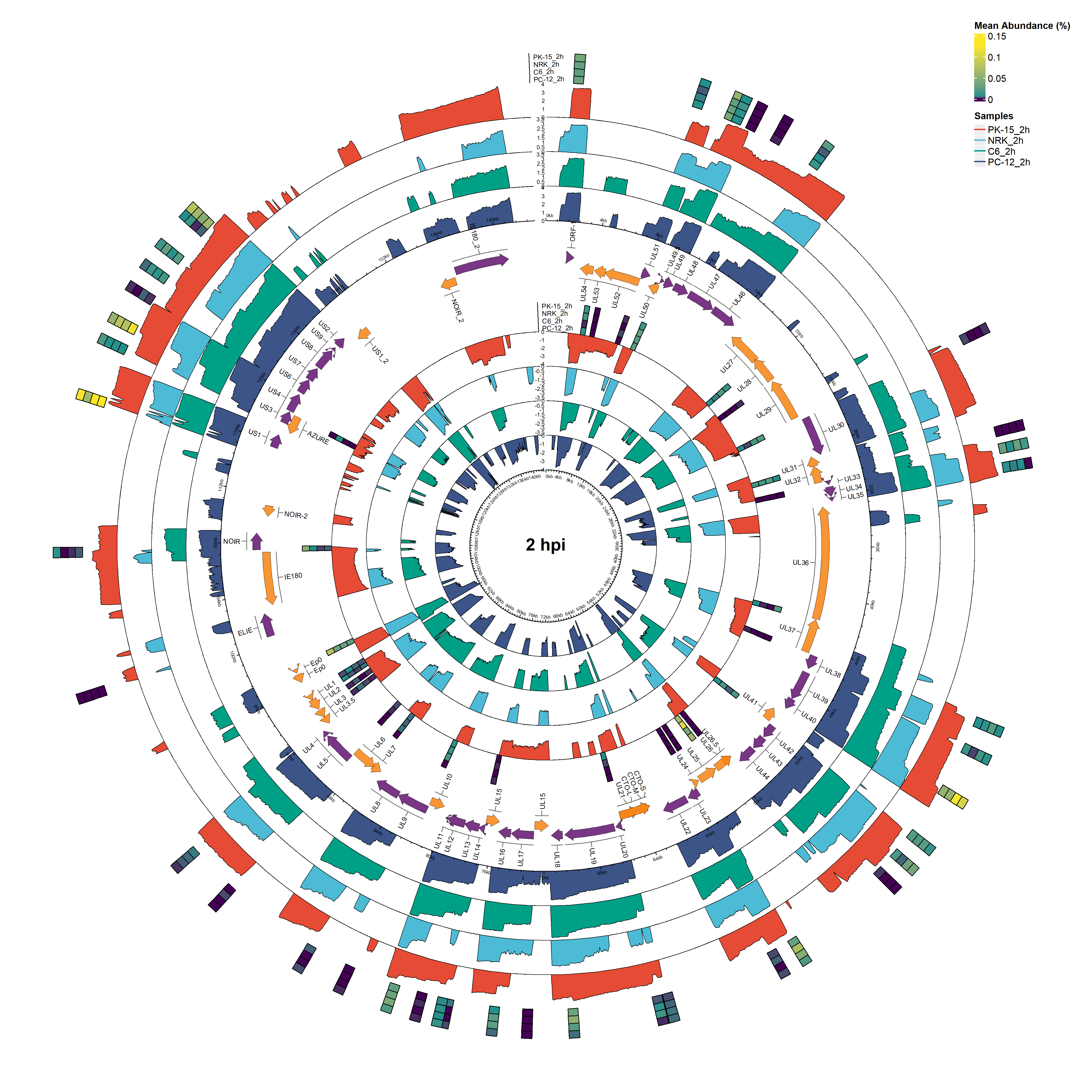

Supplement: Supplementary file 8 — Supplementary Material 8 [file 41598_2026_45990_MOESM8_ESM.png]

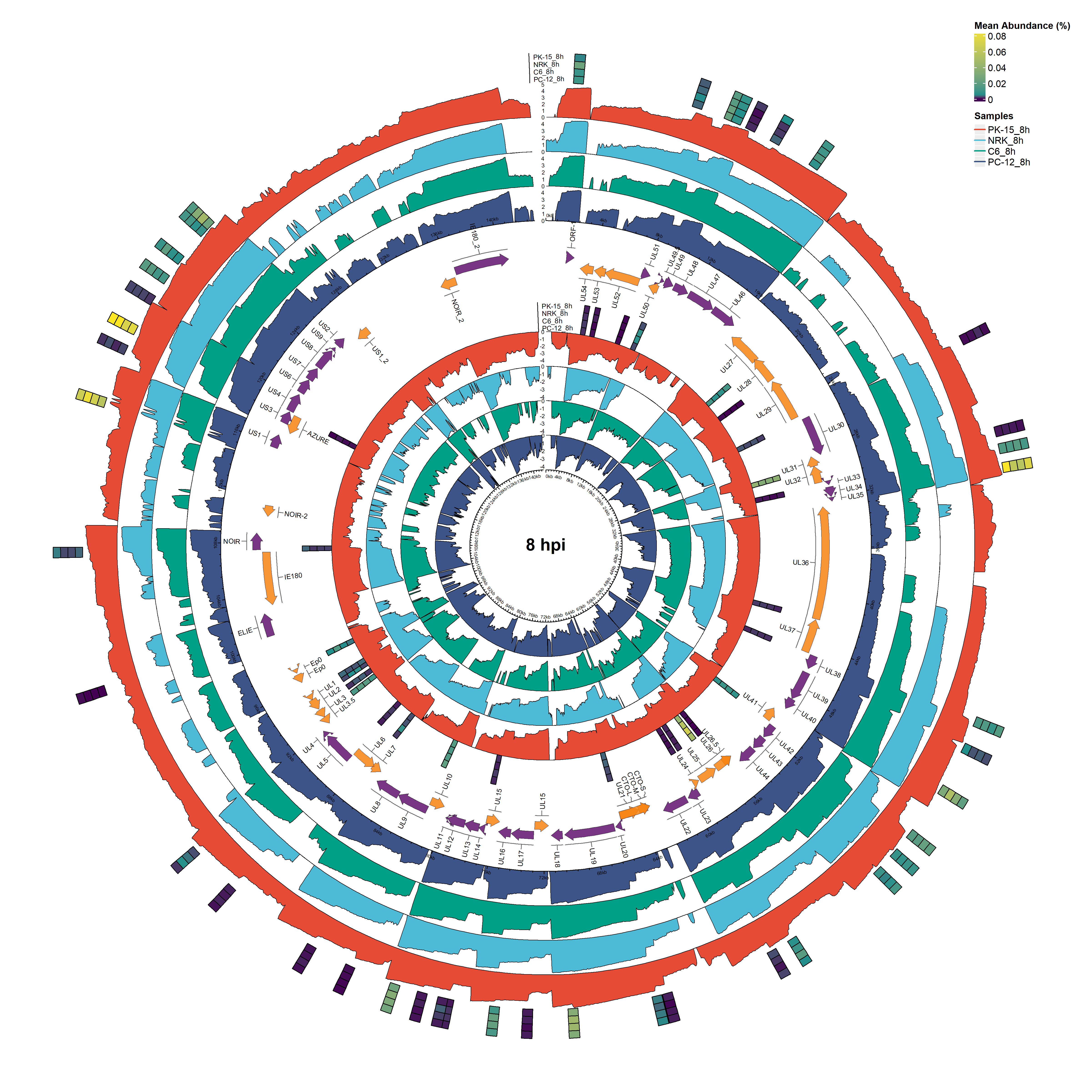

Supplement: Supplementary file 9 — Supplementary Material 9 [file 41598_2026_45990_MOESM9_ESM.png]

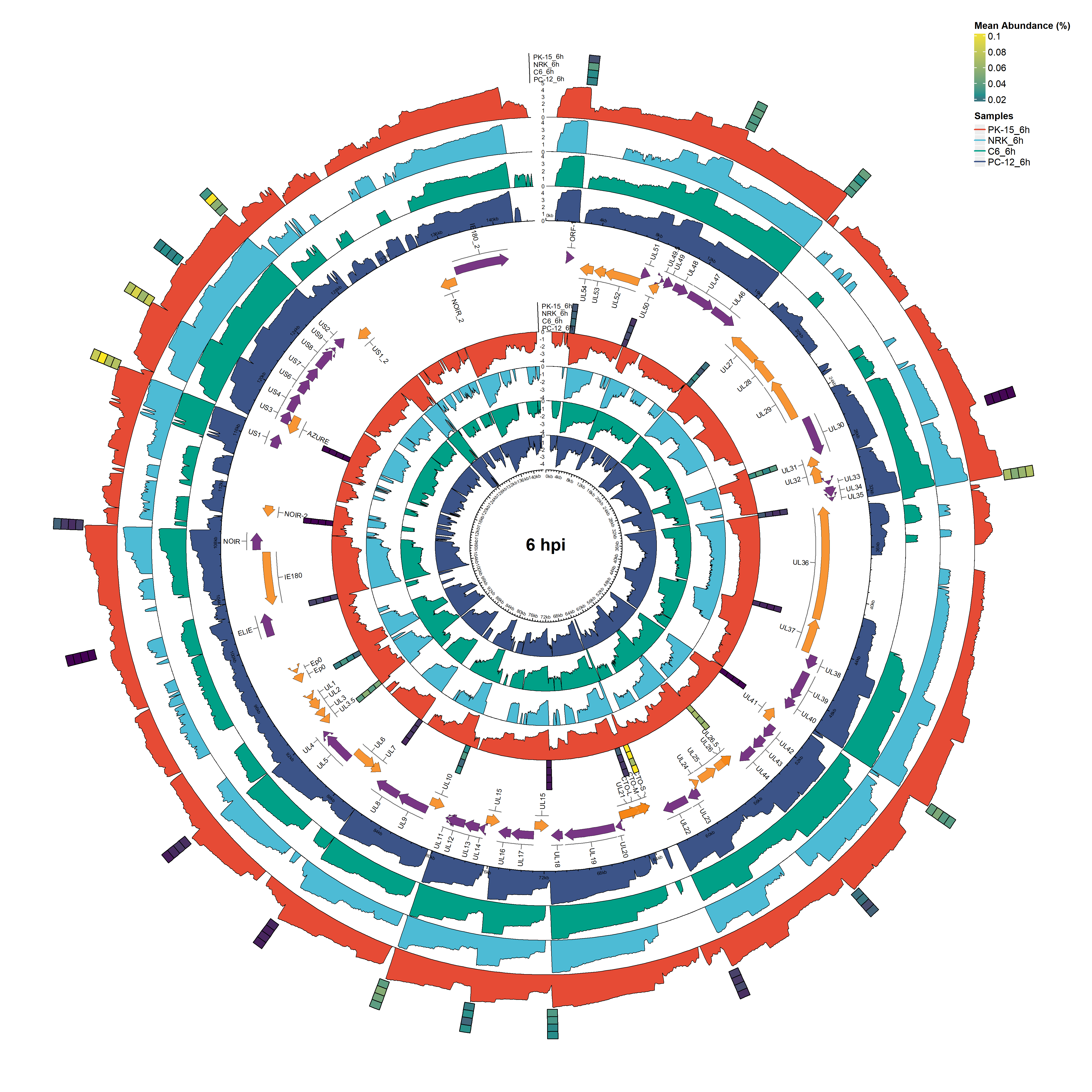

Supplement: Supplementary file 10 — Supplementary Material 10 [file 41598_2026_45990_MOESM10_ESM.png]

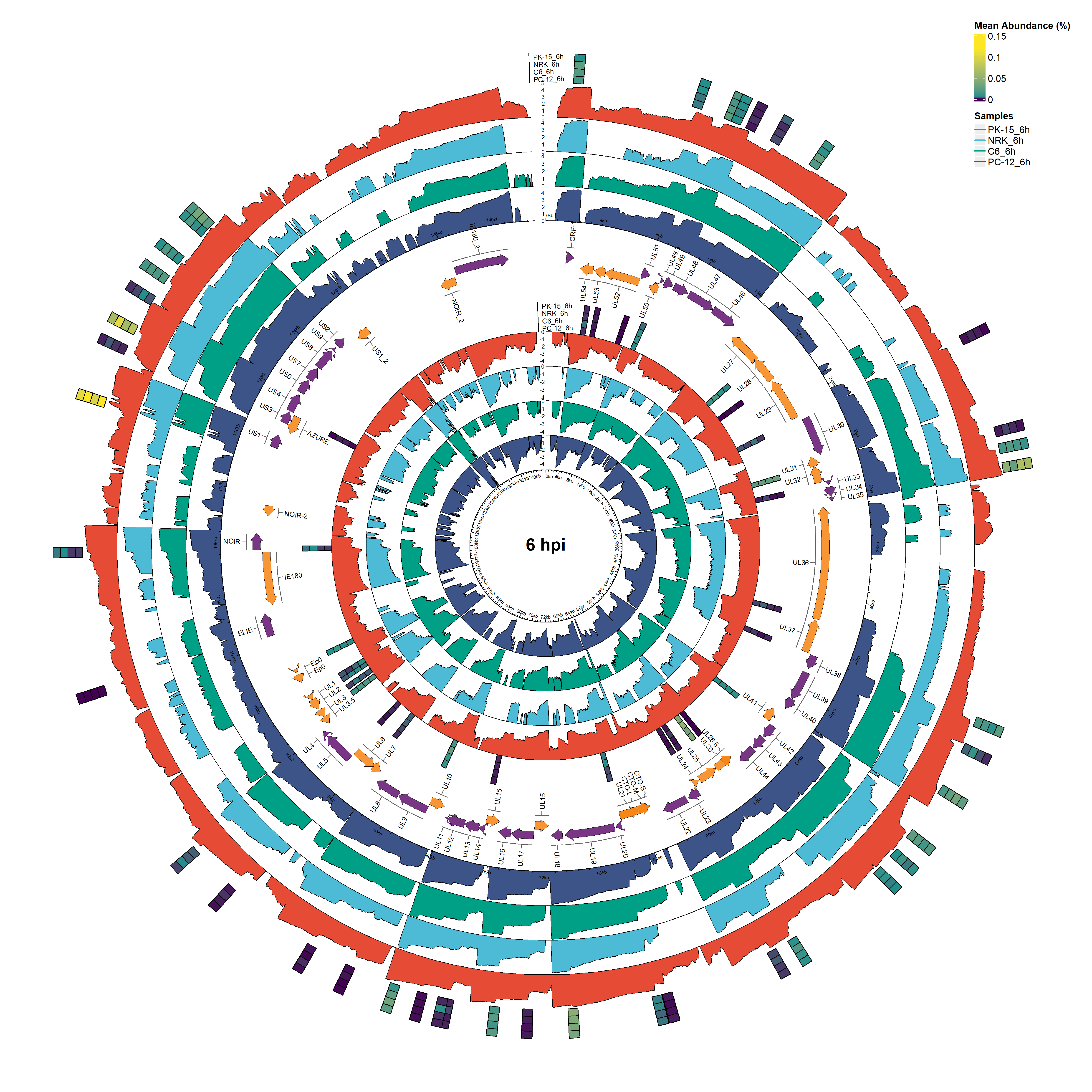

Supplement: Supplementary file 11 — Supplementary Material 11 [file 41598_2026_45990_MOESM11_ESM.png]

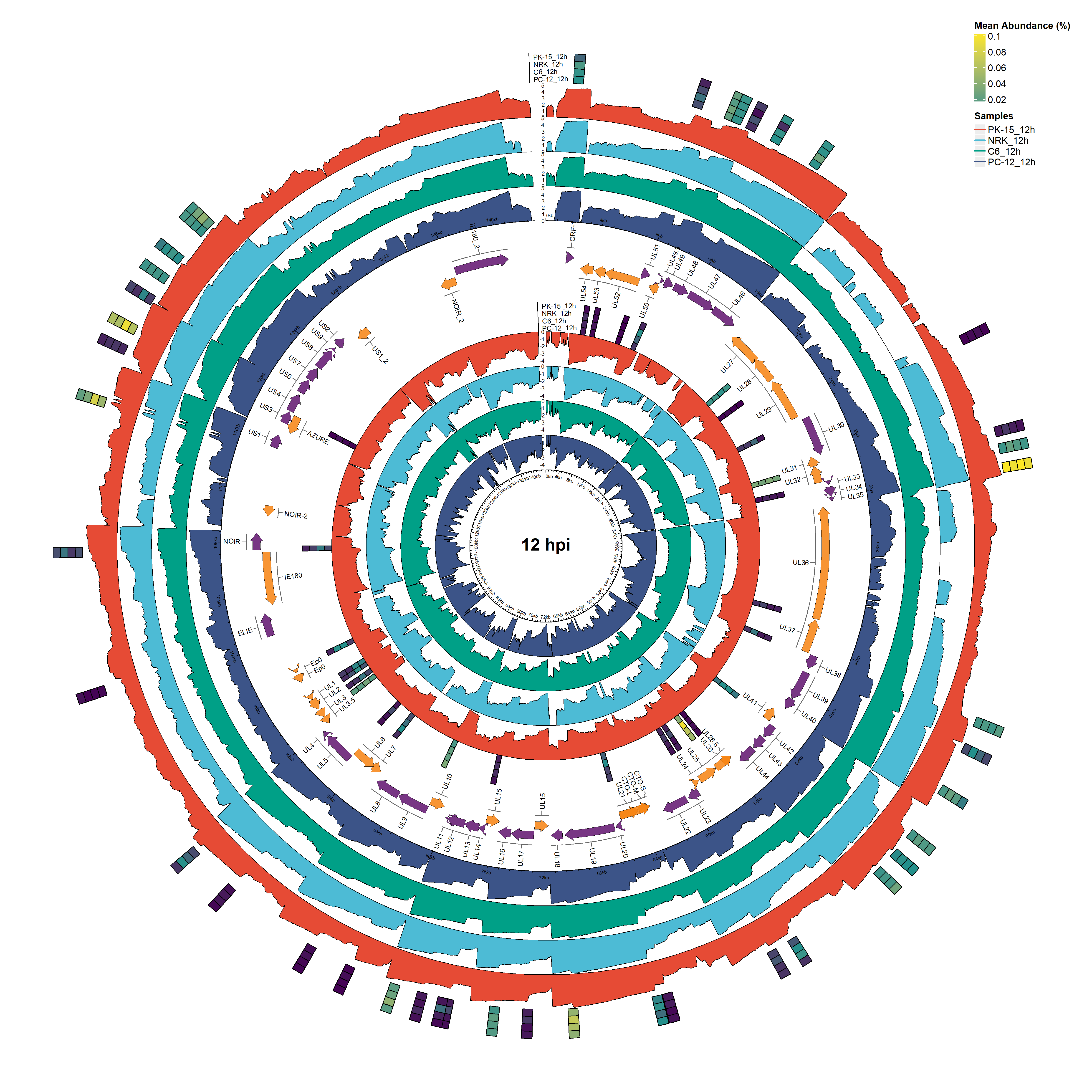

Supplement: Supplementary file 12 — Supplementary Material 12 [file 41598_2026_45990_MOESM12_ESM.png]

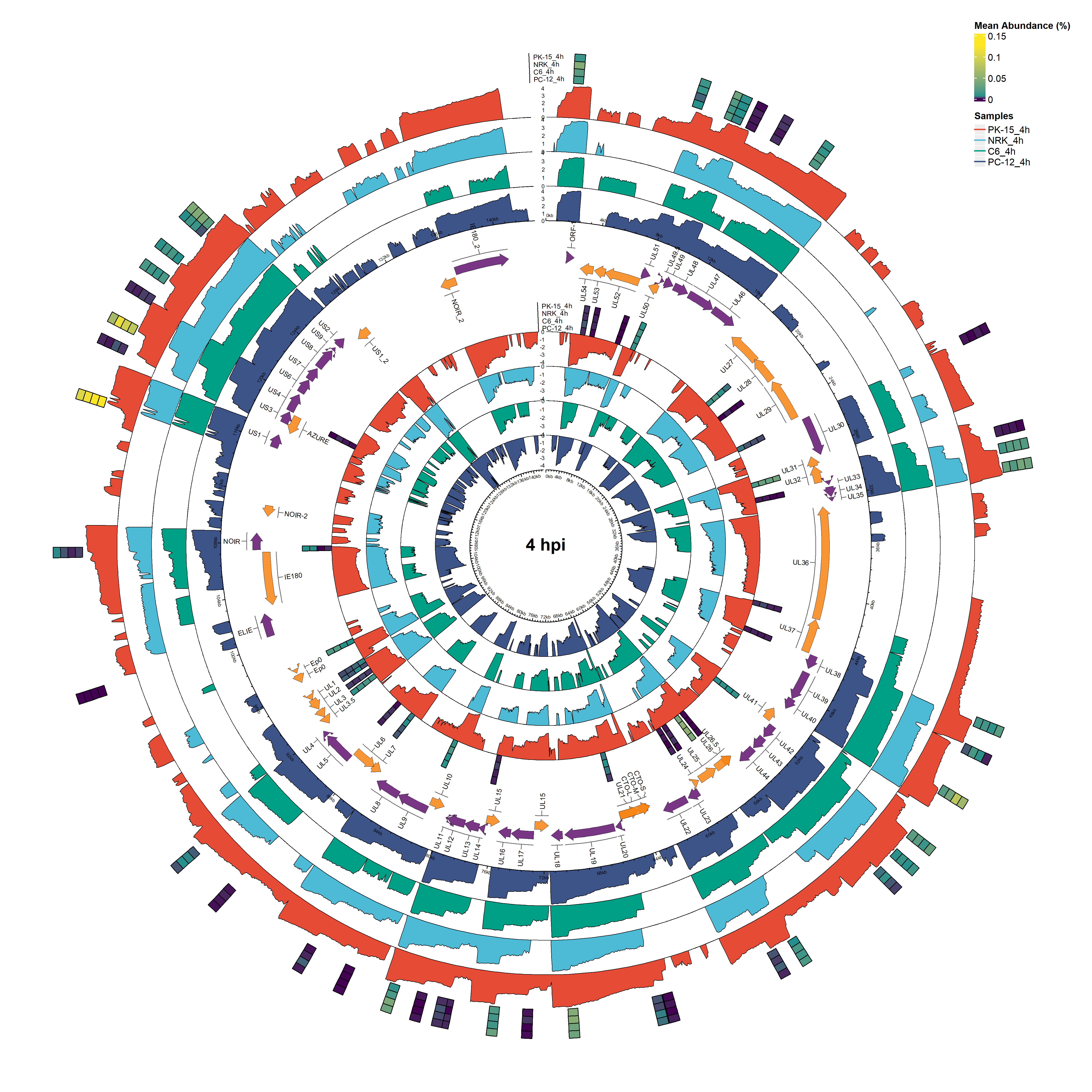

Supplement: Supplementary file 13 — Supplementary Material 13 [file 41598_2026_45990_MOESM13_ESM.png]

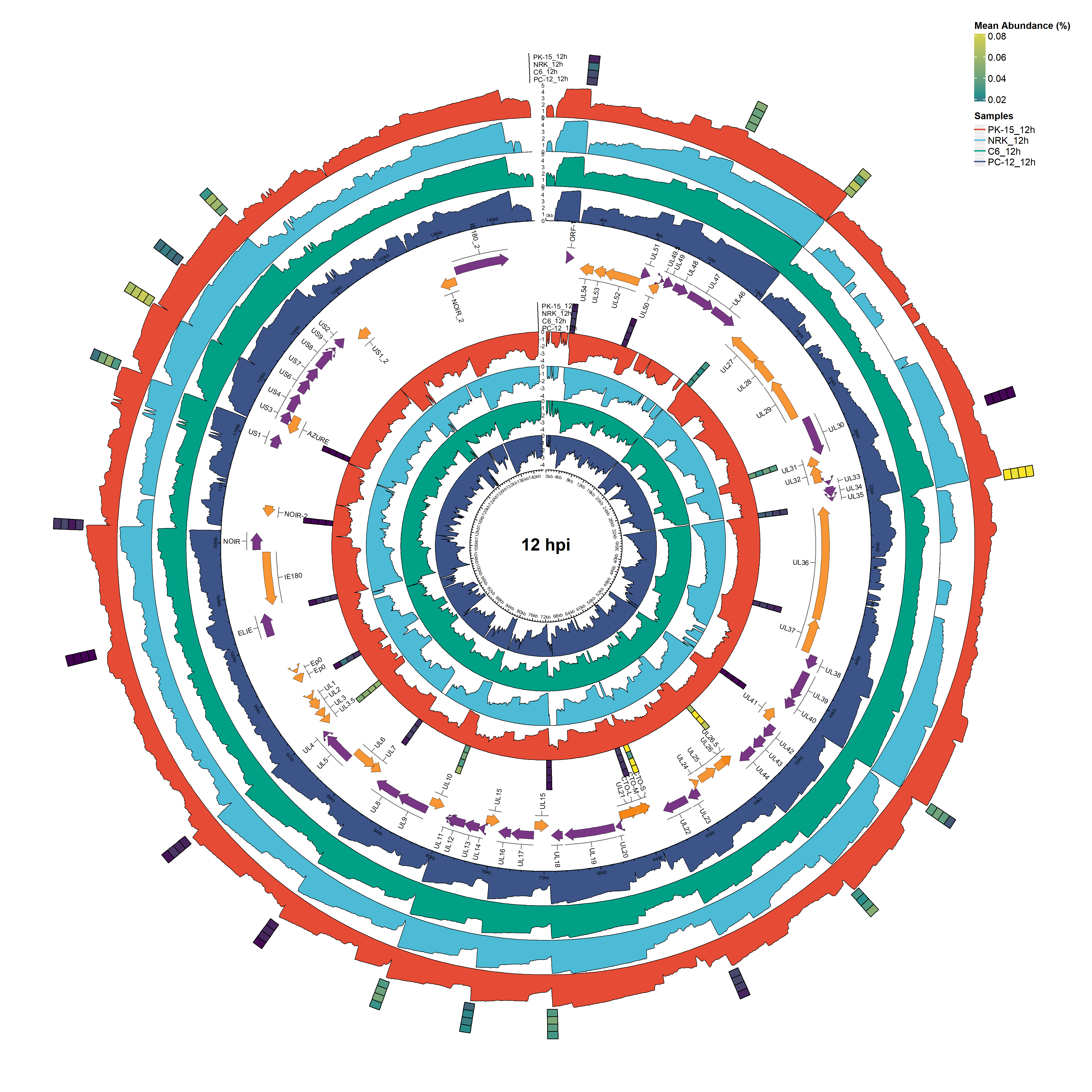

Supplement: Supplementary file 14 — Supplementary Material 14 [file 41598_2026_45990_MOESM14_ESM.png]

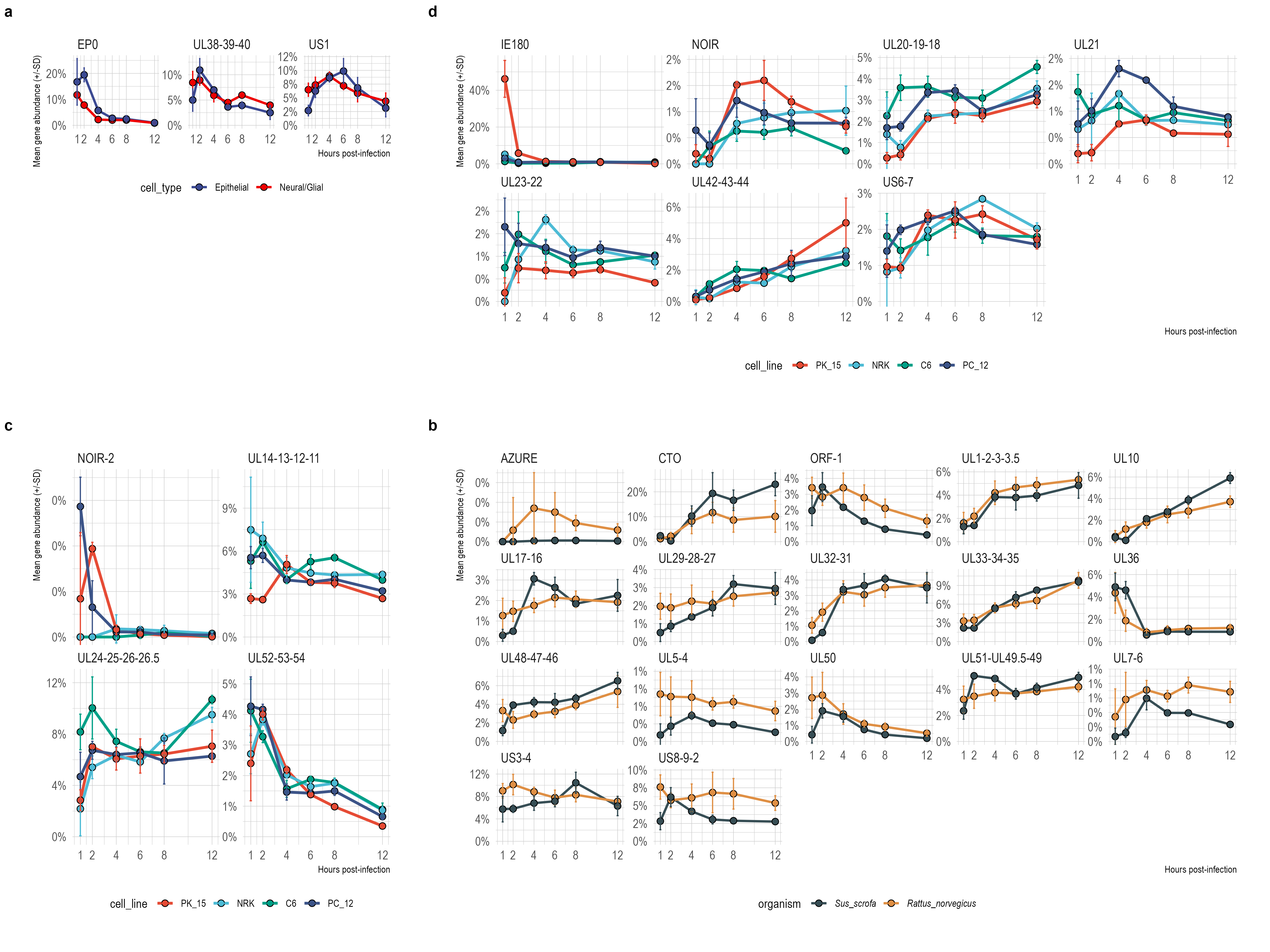

Supplement: Supplementary file 24 — Supplementary Material 24 [file 41598_2026_45990_MOESM24_ESM.png]

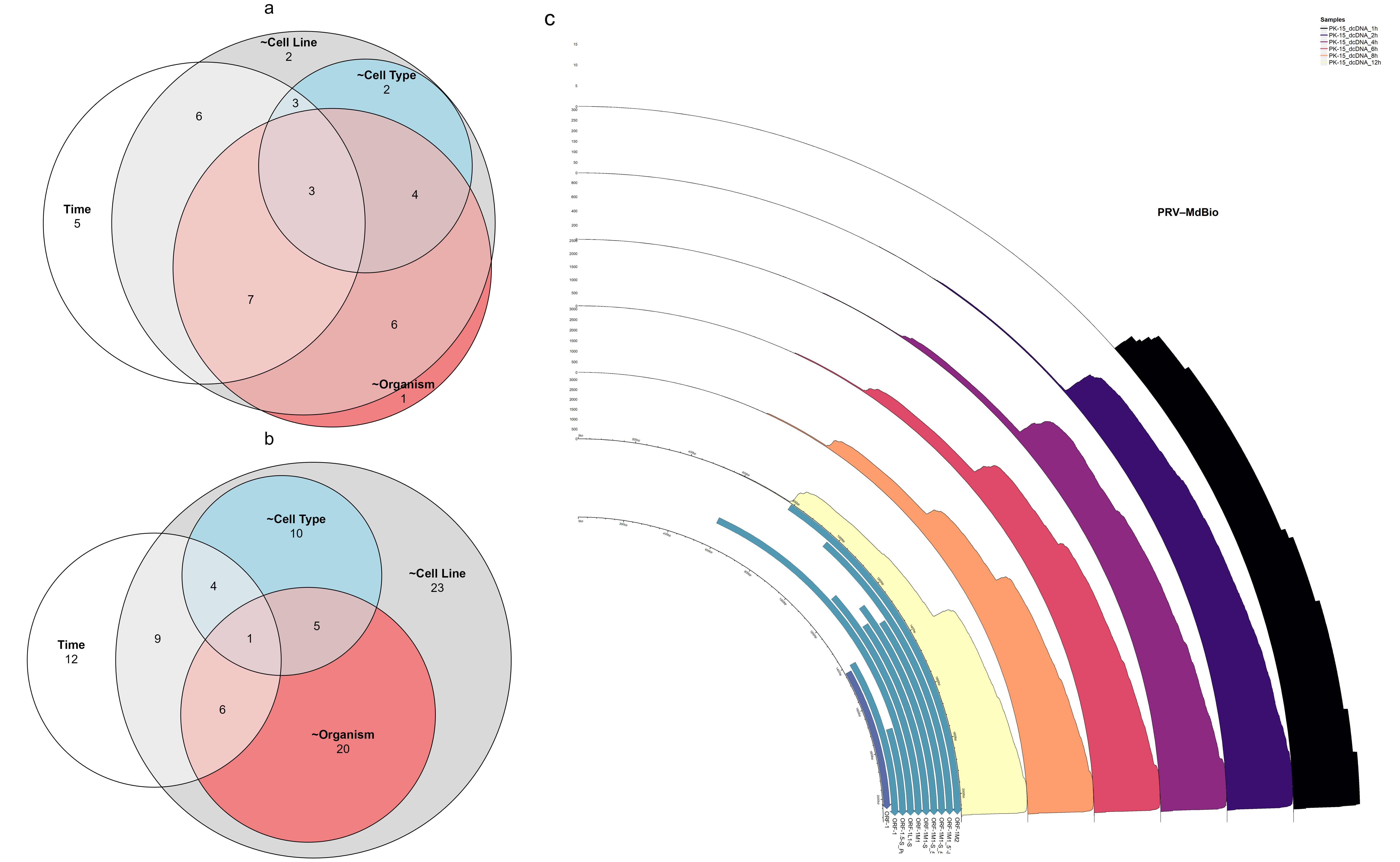

Supplement: Supplementary file 25 — Supplementary Material 25 [file 41598_2026_45990_MOESM25_ESM.png]

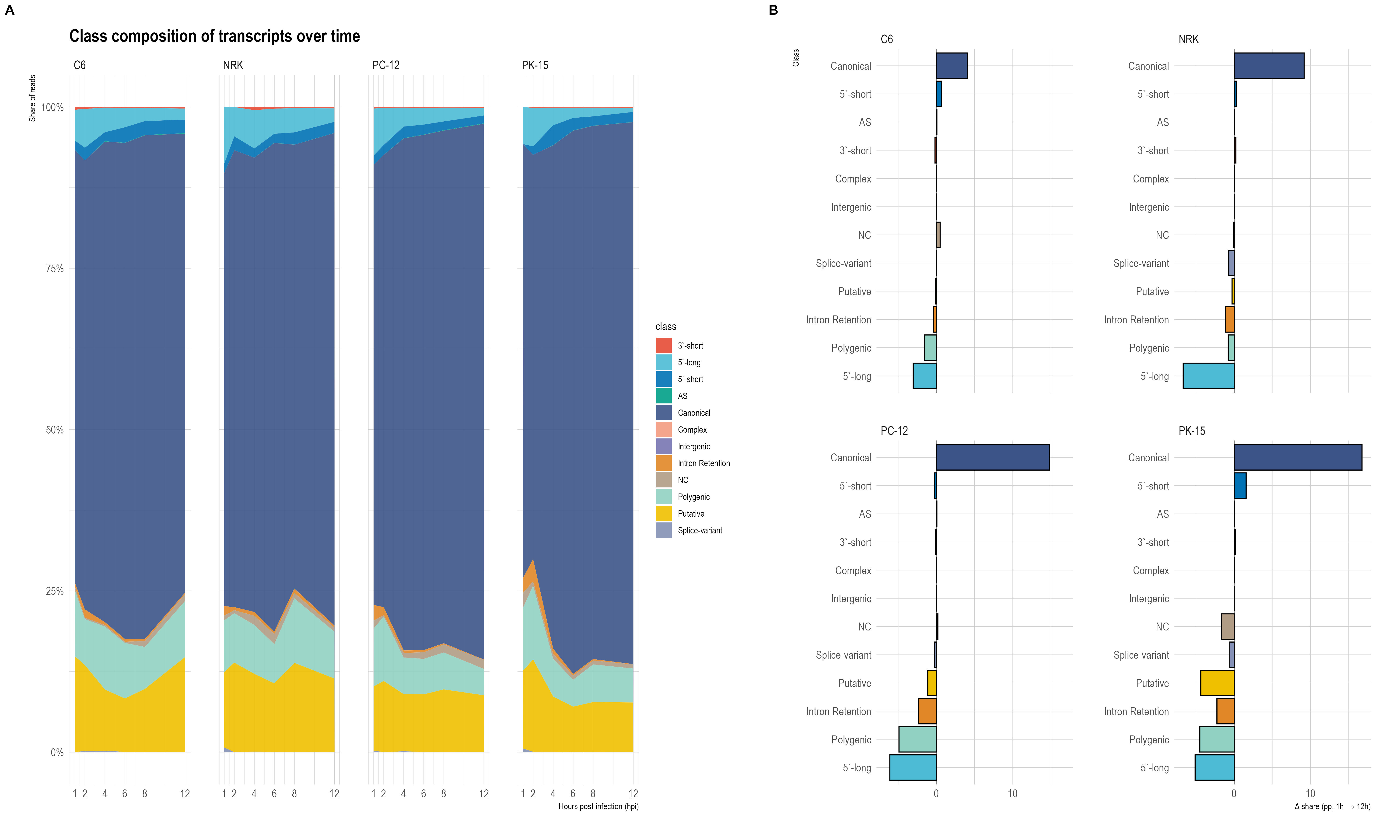

Supplement: Supplementary file 26 — Supplementary Material 26 [file 41598_2026_45990_MOESM26_ESM.jpg]
