# Supplementary material for: Multi-platform profiling reveals host- and cell -type-specific pseudorabies virus gene expression
Source: Sci Rep. 2026 Apr 1;16:15297. doi: 10.1038/s41598-026-45990-4 (PMC13181115; doi:10.1038/s41598-026-45990-4)
Supplement: Supplementary file 27 — Supplementary Material 27 [file 41598_2026_45990_MOESM27_ESM.docx]

**Legends for Supplementary Figures**

**Supplementary Figure S1. Transcript presence across cell lines and sequencing approaches.**
UpSet plot summarizing the overlap of detected PRV transcript isoforms across the four cell lines (PK-15, NRK, C6, PC-12) and sequencing approaches (dcDNA-seq time-course and mixed-time-point dRNA-seq, where available). Bars above the matrix indicate the size of each intersection, and filled dots below indicate the datasets contributing to each intersection. This analysis highlights that the vast majority of isoforms are shared across host backgrounds and library types, with only a small fraction restricted to a single cell line or sequencing approach, supporting the conclusion that host-dependent differences are predominantly quantitative rather than driven by major isoform presence/absence differences.

**Supplementary Figures S2–S7. PRV genome-wide coverage and canonical TSS abundance across time and cell lines.**

**Genome-wide strand-specific PRV coverage and canonical TSS abundance at 1, 2, 4, 6, 8, and 12 hpi in PK-15, NRK, PC-12, and C6 cells. Concentric rings display coverage as log₁₀(reads + 1). Heatmap tiles mark canonical TSS positions at their genomic coordinates and are colored according to mean abundance (percentage of viral reads per sample) using a shared color scale within each figure. Plus-strand coverage and TSS tiles are plotted on the outside, and minus-strand features on the inside. Ring order and colors correspond to the legend.**

### **Supplementary Figure S8. Cumulative transcriptional output of PRV genes based on TSS usage.**

Heatmap of total cumulative gene expression across cell lines calculated from canonical transcription start site (TSS) abundances. Cumulative expression was quantified as the area under the expression–time curve (AUC) over six infection time points (1, 2, 4, 6, 8, and 12 hpi) using the trapezoidal rule (AUC = Σ Δx × (y₁ + y₂)/2). For visualization, AUC values were square-root transformed to reduce skewness and enhance contrast. Higher values and more saturated colors indicate greater cumulative transcriptional output.

### **Supplementary Figure S9. Cumulative transcriptional output of PRV genes based on TES usage.**

Heatmap of total cumulative gene expression across cell lines calculated from canonical transcription end site (TES) abundances, computed as AUC over the full time-course (1–12 hpi) using the trapezoidal rule. AUC values were square-root transformed for visualization. This panel complements Supplementary Figure S8 by summarizing cumulative transcript completion/accumulation rather than initiation.

### **Supplementary Figure S10. Dynamics of TESs with significantly different temporal profiles.**

Line plots showing mean TES-cluster abundances (percentage of viral reads, y-axis) across the infection time-course (x-axis, hours post-infection). Each panel corresponds to coterminal gene loci (TES-defined clusters) identified as significantly differentially regulated in moanin time-course analyses. Colored lines denote cell lines: PK-15 (red), NRK (teal), C6 (green), and PC-12 (blue). Error bars indicate standard errors across biological replicates. Panels are organized by statistical intersection across additive models: (a) clusters significant exclusively in the cell-line comparison; (b) clusters significant in all three models (cell line, host species, and cell type); (c) clusters significant in the organism model (including organism-only and cell line ∩ organism intersections); and (d) clusters significant in the cell-type model (including cell-type-only and cell line ∩ cell-type intersections). This figure summarizes coterminal-locus kinetics and complements the TSS-based gene dynamics shown in **Figure 6**.

**Supplementary Figures S11–S16. Genome-wide PRV coverage and TES-based coterminal gene-cluster abundance across infection time.**

**Genome-wide strand-specific PRV coverage and TES-based coterminal gene-cluster abundance at 1, 2, 4, 6, 8, and 12 hpi in PK-15, NRK, PC-12, and C6 cells. Concentric rings display coverage as log₁₀(reads + 1). Heatmap tiles mark shared transcription end sites (TES-defined coterminal loci) at their genomic coordinates and are colored according to mean abundance (percentage of viral reads per sample) using a shared color scale within each figure. Plus-strand coverage and TES tiles are plotted on the outside, and minus-strand features on the inside. Ring order and colors correspond to the legend.**

**Supplementary Figure S17. Transcript class composition of PRV across cell lines and infection times.**

**(a) Time-resolved transcript-class composition displayed as radial stacked bar plots. Concentric rings represent successive infection time points (1–12 hpi) from the center outward. Canonical monogenic RNAs dominate across all conditions and increase further at late infection stages, while polygenic and putative classes decline over time. Early neuronal enrichment of spliced and truncated isoforms converges toward the epithelial profile by late time points. Colors denote transcript classes consistently across both panels. (b) Early-to-late differences in relative class abundance (Δ share, percentage points) between 1–4 hpi and 8–12 hpi. Each panel shows one cell line (C6, NRK, PC-12, PK-15). Canonical monogenic transcripts increase markedly over time, while polygenic and putative classes decline over time, particularly in PK-15 and NRK. Neuronal lines (C6, PC-12) display a transient enrichment of splice variants early in infection.**

**Supplementary Figure S18. Overlap of significantly altered genes and transcripts and time-resolved upregulation of ORF-1 in PK-15 cells.**

**(a, b) Euler diagrams showing the overlap between sets of significantly differentially expressed genes (a) and transcripts (b) identified in the differential expression analyses. The area of each circle and intersection is proportional to the number of significantly differentially expressed features in the corresponding set or overlap. (c) Normalized read coverage across the ORF-1 locus in PK-15 cells at successive post-infection time points, together with the associated transcript models, illustrating the progressive increase in the abundance of the putative ORF-1 transcript as infection proceeds.**

### Legends for Supplementary Tables

**Supplementary Table S1**. Read Counts in each sequencing library.

**Supplementary Table S2.** Transcript Counts across cell lines and time points.

**Supplementary Table S3.** Transcript Annotation in *gff3* format.

**Supplementary Table S4.** Relative abundance (Sheet A) and delta AUC values (Sheet B) of the regulatory triad across cell lines and time points.

**Supplementary Table S5.** Early abundances of regulatory triad transcripts in PC-12 (0.5–2.5 h). Values show mean viral read percentage ± SD (n = 3 replicates). Raw read count ranges across replicates are given in parentheses.

**Supplementary Table S6. TSS moanin model statistics**

Complete moanin time-course model results for canonical TSS-associated genes. The table reports model type (cell-line, organism, or cell-type), contrast, test level (time-course or timepoint-resolved), log2 fold-change estimates, raw p-values, and Benjamini–Hochberg adjusted q-values. Genes with q ≤ 0.05 were considered statistically significant in the main analysis.

**Supplementary Table S7. Mean TSS abundances across time points and cell lines**

Mean normalized TSS read counts for each canonical gene across all infection time points (1, 2, 4, 6, 8, and 12 hpi) and cell lines (PK-15, NRK, C6, PC-12). Values correspond to the averages used for cumulative AUC calculation and time-course modeling.

**Supplementary Table S8. Cumulative ΔAUC values for TSS comparisons**

Cumulative area-under-the-curve (AUC) values and pairwise ΔAUC differences for canonical TSS-associated genes across cell lines. ΔAUC values represent the difference between PK-15 and each comparison line (C6, NRK, PC-12). Positive values indicate higher cumulative TSS output in PK-15; negative values indicate higher output in the comparison cell line.

**Supplementary Table S9. TES moanin model statistics**

Complete moanin time-course model results for TES-associated gene clusters. The table includes model type (cell-line, organism, or cell-type), pairwise contrasts, time-course and timepoint-resolved test results, log2 fold-change estimates, raw p-values, and q-values. Clusters with q ≤ 0.05 were considered statistically significant.

**Supplementary Table S10. Mean TES cluster abundances across time points and cell lines**

Mean normalized TES cluster abundances for all coterminal gene clusters across infection time points (1–12 hpi) and host cell lines. These values form the basis for cumulative AUC calculations and comparative TES analyses.

**Supplementary Table S11. Cumulative ΔAUC values for TES comparisons**

Cumulative AUC and pairwise ΔAUC values for TES-associated gene clusters across cell lines. ΔAUC values represent PK-15 minus comparison cell line (C6, NRK, or PC-12). Positive values indicate higher cumulative TES usage in PK-15, whereas negative values indicate higher cumulative TES usage in the comparison background.

**Supplementary Table S12. Complete DRIMSeq–stageR differential transcript usage (DTU) results across all four models.**

This table contains the full per-transcript DTU output for the cell-line, cell-type, organism, and time additive models. For each transcript and contrast, the table reports model type, contrast, estimated ΔPSI (difference in percent spliced in), likelihood-ratio test statistics from DRIMSeq, raw p-values, and stage-wise adjusted p-values from stageR. Stage-wise FDR control was applied at 0.05, and transcripts with absolute ΔPSI ≥ 0.05 were considered biologically meaningful in the main analysis. All evaluated transcripts are reported regardless of significance status.
